# Down-Regulation of Hippocampal Genes Regulating Dopaminergic, GABAergic, and Glutamatergic Function Following Combined Neonatal Phencyclidine and Post-Weaning Social Isolation of Rats as a Neurodevelopmental Model for Schizophrenia

**DOI:** 10.1093/ijnp/pyw062

**Published:** 2016-07-05

**Authors:** Philip LR Gaskin, Maria Toledo-Rodriguez, Stephen PH Alexander, Kevin CF Fone

**Affiliations:** School of Life Sciences, Medical School, Queen’s Medical Centre, The University of Nottingham, United Kingdom (Drs Gaskin, Toledo-Rodriguez, Alexander, and Fone)

**Keywords:** isolation rearing, lamotrigine, microarray, phencyclidine, schizophrenia, glutamate

## Abstract

**Background:**

Dysfunction of dopaminergic, GABAergic, and glutamatergic function underlies many core symptoms of schizophrenia. Combined neonatal injection of the N-methyl-D-aspartate (NMDA) receptor antagonist, phencyclidine (PCP), and post-weaning social isolation of rats produces a behavioral syndrome with translational relevance to several core symptoms of schizophrenia. This study uses DNA microarray to characterize alterations in hippocampal neurotransmitter-related gene expression and examines the ability of the sodium channel blocker, lamotrigine, to reverse behavioral changes in this model.

**Methods:**

Fifty-four male Lister-hooded rat pups either received phencyclidine (PCP, 10mg/kg, s.c.) on post-natal days (PND) 7, 9, and 11 before being weaned on PND 23 into separate cages (isolation; PCP-SI; n = 31) or received vehicle injection and group-housing (2–4 per cage; V-GH; n = 23) from weaning. The effect of lamotrigine on locomotor activity, novel object recognition, and prepulse inhibition of acoustic startle was examined (PND 60–75) and drug-free hippocampal gene expression on PND 70.

**Results:**

Acute lamotrigine (10–15mg/kg i.p.) reversed the hyperactivity and novel object recognition impairment induced by PCP-SI but had no effect on the prepulse inhibition deficit. Microarray revealed small but significant down-regulation of hippocampal genes involved in glutamate metabolism, dopamine neurotransmission, and GABA receptor signaling and in specific schizophrenia-linked genes, including parvalbumin (PVALB) and GAD67, in PCP-SI rats, which resemble changes reported in schizophrenia.

**Conclusions:**

Findings indicate that alterations in dopamine neurotransmission, glutamate metabolism, and GABA signaling may contribute to some of the behavioral deficits observed following PCP-SI, and that lamotrigine may have some utility as an adjunctive therapy to improve certain cognitive deficits symptoms in schizophrenia.

## Introduction

Schizophrenia is a debilitating neuropsychiatric disorder with a lifetime incidence of approximately 1% worldwide. Schizophrenia is characterized by alterations in three primary symptom domains, but has no defining diagnostic neuropathology or predictive biomarkers. Positive symptoms, such as auditory and visual hallucinations and delusions, are relatively well treated with current antipsychotic medications, but negative symptoms, including avolition, anhedonia, and cognitive dysfunction, which are much less responsive to current therapy (Keefe et al., 2007), are a better predictor of therapeutic outcome (Leifker et al., 2009). Improved understanding of the neuropathology and molecular mechanisms underlying schizophrenia would enhance the probability of identifying new therapeutic targets. This process requires development and utilisation of robust, reliable rodent models for schizophrenia using behaviors with translational relevance and predictive validity (Jones et al., 2011; Millan and Bales, 2013).

Traditional rodent models for schizophrenia attempt to replicate potential aetiological factors linked to schizophrenia (e.g. neurochemical changes, hippocampal disruption, or genetic manipulation). For instance, genetic predisposition to schizophrenia has been modelled using mouse gene knock-outs, such as DISC1 and NRG1 mutants (Harrison and Law, 2006; Abazyan et al., 2010). Disrupting neurogenesis during hippocampal development with the antimitotic agent, methylazoxymethanol (Lodge and Grace, 2009), or maternal infection by administration of the viral mimetic poly(I:C) (Meyer, 2014) during pregnancy have been utilized. Early-life adversity is another established risk factor modelled using stress protocols, such as post-weaning social isolation of rodents to cause robust behavioral, neurobiological, and neurochemical deficits resembling several core symptoms in patients with schizophrenia (Fone and Porkess, 2008; Schubert et al., 2009). Illicit use of N-methyl-D-aspartate (NMDA) receptor antagonists, ketamine, and phencyclidine (PCP), produces psychosis in humans (Krystal et al., 1994) and exacerbates symptoms in schizophrenic patients (Lahti et al., 1995). Therefore, repeated NMDA receptor antagonist administration has been widely used to replicate behavioral and neurobiological changes in rodents (Wang et al., 2008; Grayson et al., 2015). In particular, neonatal PCP induces long-term locomotor abnormality, cognitive dysfunction (Depoortere et al., 2005), impaired sensorimotor gating (Wang et al., 2001) and loss of parvalbumin-containing GABAergic neurones accompanied by changes in autophagy (Radonjic et al., 2013; Jevtic et al., 2016), reminiscent of features of schizophrenia. As no single rodent paradigm completely reproduces the diverse schizophrenia symptom profile, groups have started to characterize “dual-hit” models in rodents to produce more comprehensive and robust deficits, in particular of cognitive and negative features (Ashby et al., 2010; Gilabert-Juan et al., 2012; Lim et al., 2012; Giovanoli et al., 2013; Gaskin et al., 2014; Arcego et al., 2016).

For instance, isolation rearing causes hyper-reactivity (increased ambulation) in novel arenas (Ashby et al., 2010; Hickey et al., 2012; Gaskin et al., 2014), which is enhanced by sub-chronic or neonatal NMDA receptor antagonist administration (Simpson et al., 2010; Lim et al., 2012). Moreover, neonatal PCP produces greater loss of parvalbumin-containing GABAergic interneurones (reduced in schizophrenia) than isolation rearing (Kaalund et al., 2013). Furthermore, injection of NMDA receptor antagonists such as MK-801 or PCP on post-natal days (PNDs) 7–11 followed by isolation rearing in either Sprague-Dawley or Lister-hooded rats enhances the deficit in prepulse inhibition of acoustic startle (PPI, a marker of sensori-motor gating impaired in schizophrenia) above that seen with either manipulation alone (Lim et al., 2012; Gaskin et al., 2014). Subchronic MK-801 elevated hippocampal GABA_A_ receptor expression in adult rats, coincident with isolation-induced elevations in the frontal cortex, but also increased GAT-1 expression in the frontal cortex and hippocampus (Hickey et al., 2012), not reported with the latter. Thus neonatal NMDA antagonist administration may induce glutamatergic hypofunction, thought to contribute to negative and cognitive deficits in schizophrenia not reproduced by isolation alone.

Current antipsychotics are high-affinity dopamine D_2_ receptor antagonists, which is thought to account for their effectiveness against positive symptoms but have limited benefit against negative and cognitive deficits of schizophrenia. Therefore, new adjunctive therapeutics operating on novel pharmacological targets are essential to improve treatment.

The anticonvulsant drug lamotrigine (prescribed to treat epilepsy and bipolar disorder) blocks voltage-gated sodium channels (Na_v_1.2), preventing neuronal depolarization and reducing synaptic release of excitatory amino acids, including glutamate and aspartate (Leach et al., 1991). In addition, lamotrigine has weak inhibitory effects at the 5-HT_3_, 5-HT_1A_, and nicotinic acetylcholine receptors (Bourin et al., 2005; Zheng et al., 2010). Although not approved to treat schizophrenia, clinical studies have noted lamotrigine may improve some symptoms in a clozapine-resistant patient population (Goff, 2009; Tiihonen et al., 2009). Notably, lamotrigine and related voltage-gated sodium channel blockers attenuate cognitive deficits in methamphetamine- and PCP-treated rats and transgenic mice models used to investigate schizophrenia, including visual and spatial learning (Celikyurt et al., 2012), reversal learning (Large et al., 2011), and PPI deficits (Brody et al., 2003b; Nakato et al., 2010). The mechanisms underlying these pro-cognitive effects are unclear but could involve modulation of cortical glutamate release. Furthermore, as risperidone also blocks voltage-gated sodium channels it is plausible that this mechanism might also underlie some of the beneficial effects of this antipsychotic (Brauner et al., 2014).

This study characterizes alterations in neurotransmitter signaling pathways (in particular dopamine, glutamate, and GABA) using microarray and examines the ability of lamotrigine to reserve behavioral deficits in rats given combined neonatal PCP and isolation rearing, an established neurodevelopmental model for schizophrenia. Our aim was to compare changes in hippocampal gene expression with those reported in schizophrenia and our prediction was that lamotrigine would reverse some of the cognitive deficits produced by this rodent model for schizophrenia.

## Methods

### Animals

Eight litters of three-day old, male Lister hooded rat pups (n = 54) were obtained from Charles River UK, accompanied by their natural dam. On PNDs 7, 9, and 11, half of the pups from each litter were treated with phencyclidine hydrochloride (PCP; Sigma-Aldrich) dissolved in 0.154M sterile saline (10mg/kg s.c.) and the rest were treated with saline (1ml/kg), as described previously (Gaskin et al., 2014; Watson et al., 2016). To avoid maternal rejection of pups, hands were washed and rubbed in bedding material prior to injection and photographic records of pelage were used for identification and to avoid repeated marking. Pups were monitored for 3h following injection and only disturbed to return to the nest if separated from the dam. Pups were weaned by litter on PND 23, and those receiving saline were housed in groups of 3–4 (32 x 51cm polycarbonate cages with metal grid lids, V-GH, n = 23), while PCP-treated pups were housed alone in social isolation (25 x 42cm cages, PCP-SI, n = 31) for the entire study. V-GH and PCP-SI rats were housed in the same holding room, enabling visual, auditory, and olfactory contact. Cages contained sawdust bedding (but no environmental enrichment) changed once per week when each rat was weighed, but rats were otherwise undisturbed until behavioral testing commenced five weeks later. Rats had free access to water and food (BeeKay Standard Laboratory Diet, BioSystems), and were maintained on a 12h light dark cycle (lights on 0700h) in controlled humidity (45±15%). Rats were divided into two groups to examine the impact of lamotrigine on behavior (V-GH, n = 15; PCP-SI, n = 23), or the effect of developmental manipulation (PCP-SI) on gene expression by microarray. In the microarray study rats only underwent locomotor assessment (to confirm development of the isolation syndrome) to prevent further behavioral assessment affecting microarray data (V-GH, n = 8; PCP-SI, n = 8). In both groups, natural brothers were paired for treatment, to minimize the impact of genetic variation on outcome. All procedures were carried out in accordance with the Animals (Scientific Procedures) Act, 1986, with University of Nottingham ethical committee approval and in accordance with the ARRIVE guidelines.

### Experimental Design and Drugs

All studies were performed on rats that received both neonatal PCP and subsequent social isolation or vehicle-treated group-housed littermates. The independent impact of exposure to each of these two developmental manipulations has been thoroughly studied before (for reviews see Fone and Porkess [2008] and Grayson et al. [2015]) and compared with the combined treatment by us (Gaskin et al., 2014; Watson et al., 2016). The current study was designed to compare the ability of lamotrigine to reverse the resultant behavioral syndrome but not to determine which alterations were produced by either neonatal PCP or isolation alone. To reduce unnecessary use of animals, in accordance with the 3Rs principle and the ARRIVE guidelines, separate groups (PCP or isolation rearing alone) were not included in this study. To ascertain any potential confounding motor effect of lamotrigine, indices of locomotor activity were carefully monitored in each behavioral task and findings are discussed later. Lamotrigine (Sigma-Aldrich) was suspended in 50% w/v methylcellulose, containing 1M HCl adjusted to pH 7.4 with 0.1M NaOH. Prior to behavioral testing, rats were divided into lamotrigine or control groups by drawing lots, such that PCP-isolation-reared rats (hereafter referred to as PCP-SI) received either vehicle (2ml/kg, PCP-SI-V, n = 8) or a low dose (10mg/kg, PCP-SI-L10, n = 8) or high dose (15mg/kg, PCP-SI-L15, n = 7) of lamotrigine and group-housed perinatal saline-treated (hereafter V-GH) rats received either vehicle (V-GH-V, n = 8) or a high dose of lamotrigine (15mg/kg, V-GH-L15, n = 7). All treatments were administered by intraperitoneal injection (2ml/kg) 60min before each behavioral test. Doses of lamotrigine were selected from previous rat cognitive behavioral studies (Brody et al., 2003b; Celikyurt et al., 2012).

### Behavioral Testing

Behavior was examined (between 0900 to 1600h) in a battery of tasks selected for translational relevance to positive (locomotor activity in a novel arena) and cognitive (novel object recognition [NOR] and PPI) symptoms of schizophrenia, in order of least to most aversive to minimize the impact of the previous test on the subsequent assessment. In all cases rats were acclimatized to the behavioral room for at least 30min before testing, the apparatus was cleaned between each use with 20% alcohol, and the observer was unaware of drug treatment. Behavioral observations commenced on PND 63 using identical protocols to those described previously (King et al., 2004; Schubert et al., 2009; Watson et al., 2012, 2016; Gaskin et al., 2014).

### Locomotor Activity

In order to assess the exploratory response to a novel arena, which is recognized as a valid marker for positive symptoms sensitive to antipsychotic treatment (Fabricius et al., 2010; Jones et al., 2011; McIntosh et al., 2013), open field locomotor exploratory activity (LMA) was recorded in infrared activity chambers (clear Perspex boxes 39 x 23.5 x 24.5cm). Rats were assigned individual chambers crossed by infrared beams (Photobeam Activity System, San Diego Instruments) to measure horizontal ambulatory movements as number of beams broken in 10min bins for 1h (Bianchi et al., 2006; Jones et al., 2011).

### Novel Object Recognition

To assess visual learning and memory, a simple two-trial NOR discrimination task with established translational relevance to visual recognition memory impaired in schizophrenia (Lyon et al., 2012; Rajagopal et al., 2014) was used 24h after locomotor assessment. Each rat being assigned to the same chamber previously used. Rats were placed in their chamber for 3min acclimatisation, before returning to their home cage for 1min to enable two identical objects (8cm high plastic bottles, 5cm diameter, filled with water and secured to the floor by blue-tac) to be introduced. Rats were then returned to chamber, and exploration of each object recorded separately for 3min (familiarization, trial 1). For 2h rats were returned to their home cage, and one object selected in a pseudo-random manner replaced with a visually distinct novel object (identical size bottle covered in three rings of 2cm black tape). During the second 3min choice trial (trial 2) object exploration, directed attention to each object with the nose ≤1cm away (accompanied by sniffing with active vibrissae), was recorded using stop watches. Climbing on or chewing the object was not recorded as active exploration.

In addition to comparing the actual time (s) exploring the novel and familiar objects in the choice trial the discrimination ratio (D2) was calculated [novel object exploration/(novel + familiar) object exploration time] as an index of preferential object exploration independent of total exploration time.

### Prepulse Inhibition of Acoustic Startle

To assess changes in sensorimotor gating, which shares a neurobiological equivalence and is thought to map to the pre-attentional processing cognitive domain in humans, rats were examined in a prepulse inhibition of acoustic startle test. PPI was recorded in computerized startle boxes (San Diego Instruments) as described previously (Schubert et al., 2009; Jones et al., 2011). After acclimatization to white background noise (65 dB, 5 mins), rats received 66 x 120 dB tones (40ms in duration) either delivered alone or preceded by a 20ms prepulse of 72, 76, 80, or 84 dB with an unpredictable inter-trial interval (10–20s). The startle amplitude to tone alone (first ten and last ten startles) was compared to calculate habituation, and used to calculate the average percentage inhibition of startle (%PPI) for each prepulse-pulse combination. Previous studies by our group have shown that startle responses to 72 dB trials fail to significantly attenuate PPI, but exclusion of these trials leads to greater variation in response to other prepulse intensities, so the data from 72 dB prepulse trials were collected, but excluded from analysis. A conditional statement was included in the analysis software to automatically exclude any trial whose %PPI was >2 standard deviations from the mean, which could occur due to rat movement during startle exposure.

### RNA Isolation for DNA Microarray

On PND 70, following locomotor activity assessment (see above) rats in the microarray study were culled by concussion followed by immediate decapitation to collect the whole left hippocampus, which was frozen in liquid nitrogen in RNAse-free tubes (Corning). Using sterile dissection, RNA was isolated using TRI Reagent (Sigma-Aldrich). Sample concentration and purity was tested using nanodrop and Bioanalyser 2100 (Agilent) and 14 samples (two rats with low RNA yield were excluded) were selected from three litters, divided so natural brothers were paired across the two treatment groups to minimize the impact of natural genetic variation on analysis. RNA samples were hybridized onto Affymetrix GeneChip Rat Exon Gene 1.0 ST microarrays (Affymetrix) and subsequently analyzed at the Hospital for Sick Children in Toronto, Canada.

Prior to conducting the microarray analysis, published literature was used to identify a list of candidate genes based on their known biological, neurophysiological, or functional relevance to schizophrenia, with the knowledge that any of these showing the greatest differential expression from microarray analysis would be selected for further Q-PCR analysis. As this approach would limit analysis to the existing theoretical neurobiology of the disorder, we also planned to select genes with the highest differential expression that were in key regulatory positions in any canonical pathways also identified as affected. Microarray data was analyzed to determine change in expression meeting lenient critical thresholds of a combined change in expression (*p* < 0.05) and a q-value <55%. Genes meeting criteria were: (i) entered into the Ingenuity Pathway Analysis software (IPA, Ingenuity Systems Inc., QIAGEN) for further investigation and (ii) selected for subsequent analysis by quantitative PCR. For this purpose, total RNA was reverse transcribed with SuperScript III and random primers (Promega UK). Q-PCR reactions were performed in triplicate with the SensiMix Plus SYBR Green PCR kit (Bioline) and a Rotor Gene 3000 cycler (Corbett Life Science, Qiagen UK). Q-PCR primers were designed with Primer3 (www.genome.wi.mit.edu/cgi-bin/primer/primer3.cgi) and optimized in-house (Table 1) both for the genes of interest and control housekeeping genes (HPRT1, PGK1, GAPDH).

**Table 1.** Primer Sequences for Q-PCR Analysis

Gene Name	Forward Primer	Reverse Primer	Product Size (bp)
**Housekeeping Genes**			
Hprt1	cgaggagtcctgttgatgttgc	ctggcctataggctcatagtgc	172
Pgk1	tagtggctgagatgtggcacag	gctcacttcctttctcaggcag	166
GAPDH	ggcaagttcaatggcacagt	tggtgaagacgccagtagactc	183
**Genes of Interest from Microarray**			
DDC	gcctgattcctttcttcgtg	acgccattcagaagataccg	175
DRD5	agacacggtcttccacaagg	cacagtcaagctcccagaca	185
GABBR1	caagagcgtgtccactgaaa	gcacaaagagcacaaccaga	191
GABRB2	ctgggtctccttttggatca	ccagaagggccataaagaca	185
GABRA4	ggacagtttgctggatggtt	tggggccatcatatttcagt	185
GAD1	cacaaactcagcggcataga	gccttgtcccctgtatcgta	194
GAT	ggggccttcctaattccata	gtagatggcccaggagatga	210
PVALB	gagtgcggatgatgtgaaga	gtcagcgccacttagctttc	228

### Statistical Analysis

Microsoft Excel 2007, GraphPad Prism v6 (GraphPad Software Inc.) and InVivoStat were used for statistical analyses. Data were checked for normality and homogeneity of variance using Shapiro-Wilk’s and Levene’s tests, respectively. LMA, NOR, and PPI data were analyzed by three-way repeated measures analysis of variance (RM ANOVA, with rearing condition and drug challenge as main factors, and time, object, or pre-pulse volume as the repeated measure). Choice trial discrimination ratio was analyzed by two-way ANOVA (with pre-exposure and drug challenge as factors). In each case, where appropriate, multiple comparison post hoc tests were used where ANOVA suggested statistical significance (considered *p* < 0.05). Q-PCR results were analyzed using REST 2009 software (QIAGEN and Technical University) to give relative fold-changes in gene expression and a *p*-value confidence interval. All data are presented as mean ± SEM and *p* < 0.05 was considered significant.

## Results

### Effect of Lamotrigine on PCP-SI-Induced Changes in Locomotor Activity

**Figure 1.**
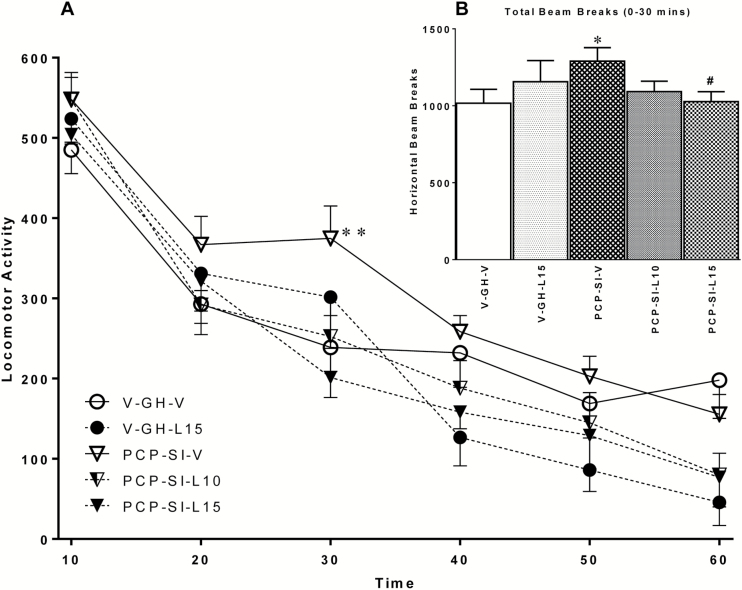
Lamotrigine attenuated PCP-SI rearing induced hyperlocomotion in a novel arena, without reducing horizontal activity in V-GH controls. (A) Locomotor beam breaks (mean ± SEM in consecutive 10min epochs, n = 7–8) significantly decreased over 60min [F_(11,352)_ = 93.408, *p* < 0.001, RM ANOVA], reflecting habituation to a novel arena. Rearing in social isolation (PCP-SI) significantly increased activity compared to group housed (V-GH) controls [F_(1,32)_ = 4.570, *p* = 0.040, RM ANOVA], which was reversed by lamotrigine at 15mg/kg i.p. (L15) but not 10mg/kg i.p. [L10; housing x drug interaction, F_(1,31)_ = 5.501, *p* = 0.025, RM ANOVA]. **p < 0.01 from PCP-SI-L15 lamotrigine, Bonferroni’s post hoc following ANOVA. PCP-SI, phencyclidine-treated pups housed alone in social isolation.

As expected, when placed in a novel arena, horizontal activity gradually decreased in all groups, reflecting habituation to the mildly aversive environment, supported by a significant main effect of time [F_(11,352)_ = 93.408, *p* < 0.001]. Isolation-reared rats were significantly more active than group-reared counterparts, reflected by a significant main effect of housing condition [F_(1,32)_ = 4.570, *p* = 0.040]. Despite there being no overall significant effect of lamotrigine on locomotor activity [F_(2,31)_ = 2.985, *p* = 0.065], there was a significant housing x drug interaction [F_(1,31)_ = 5.501, *p* = 0.025] over the 60min session, reflecting a reduction in activity due to lamotrigine treatment in the isolation-reared animals only (Figure 1A). Furthermore, two-way ANOVA of total activity during the first 30min (where activity differences were most marked, as often found in isolation studies; Bianchi et al., 2006; Fabricius et al., 2010; McIntosh et al., 2013) revealed a significant rearing x drug interaction [F_(1,32)_ = 6.719, *p* = 0.014], but no main effect of either rearing condition or drug treatment alone (Figure 1B). Of note, post hoc analysis confirmed there was a significant increase (*p* < 0.05) in locomotion in PCP-SI-V compared to control V-GH-V rats, which was significantly reduced (*p* < 0.05) by the highest dose of lamotrigine (PCP-SI-L15) compared to that in PCP-SI-V rats. Taken together, this suggests isolation rearing induced mild hyperactivity that was partially reversed by lamotrigine, most notably at the highest dose.

(B) Total locomotor counts during the first 30min were elevated in PCP-SI rats and were significantly attenuated by lamotrigine (15mg/kg), without any accompanying decrease in locomotion in V-GH-L15 rats treated with the same dose. *p < 0.05 from V-GH-V controls and #*p* < 0.05 from PCP-SI-V rats, Bonferroni’s post hoc test following ANOVA.

To confirm development of the isolation syndrome in rats utilized for microarray analysis, PCP-SI reared rats in this study also displayed significantly elevated LMA (*p* = 0.0275) compared to V-GH controls in the activity chambers (data not shown).

### Effect of Lamotrigine on PCP-SI-Induced Deficits in Novel Object Recognition

**Figure 2.**
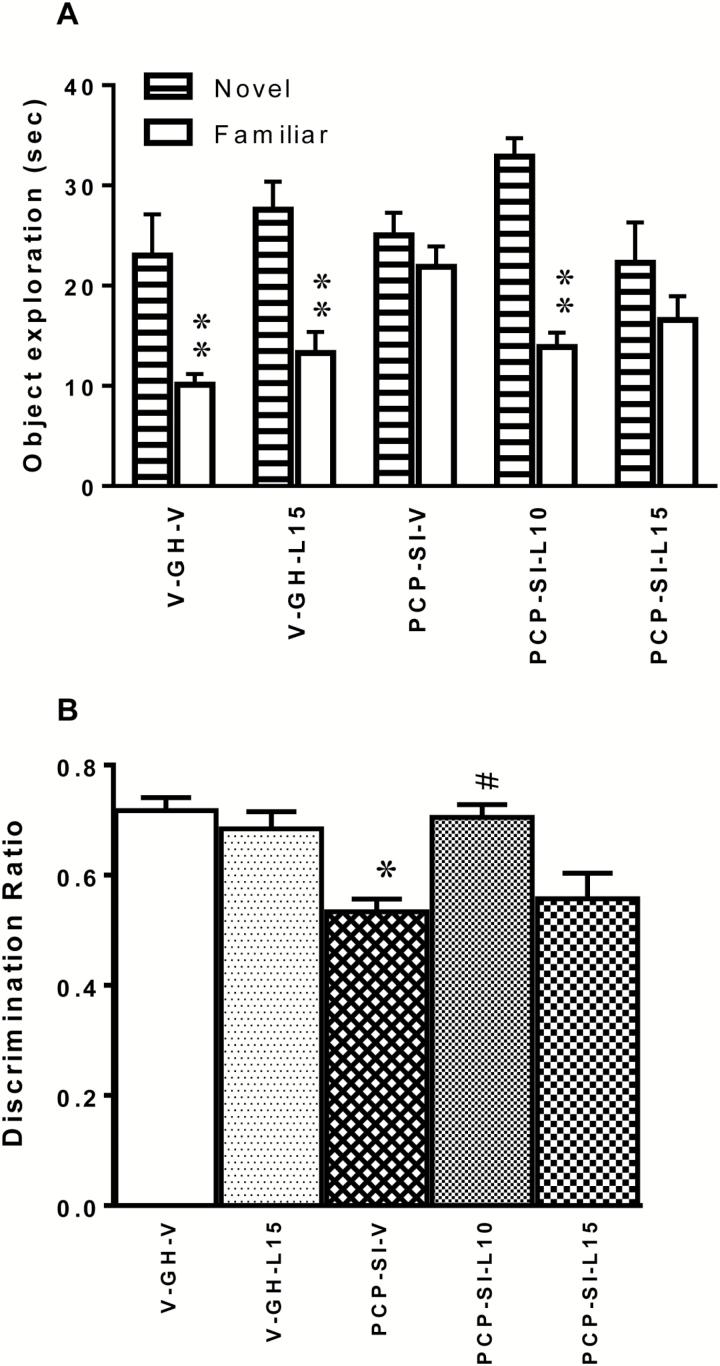
Impairment in novel object recognition (NOR) discrimination following PCP-SI rearing was reversed by lamotrigine treatment at 10mg/kg. (A) Irrespective of whether they received acute treatment with 15mg/kg lamotrigine (V-GH-L15) or vehicle (V-GH-V), group-housed rats preferentially explored the novel over the familiar object (s, mean ± SEM, n = 7–8) during the second choice trial during the NOR task [significant object x treatment interaction by two-way ANOVA, F_(4,29)_ = 4.620, *p* = 0.0045]. While vehicle-treated PCP-isolation reared rats (PCP-SI-V) could spent an equal time exploring both objects, discrimination of the novel object was restored following acute treatment with the highest dose (15mg/kg) of lamotrigine (PCP-SI-L15). PCP-SI-reared rats receiving 10mg/kg lamotrigine (PCP-SI-L10) were able to discriminate between the two objects. **p < 0.001 novel vs familiar, Bonferroni’s post-hoc test following ANOVA. PCP-SI, phencyclidine-treated pups housed alone in social isolation.

To determine any impact of neurodevelopmental manipulation or acute drug treatment on visual learning and memory, rats were examined in the NOR paradigm. During the second (choice) trial, ANOVA showed a main effect of object [F_(1,32)_ = 64.72, *p* < 0.0001] and a significant object x treatment interaction [F_(4,29)_ = 4.620, *p* = 0.0045], but no main effect of drug treatment [F_(4,29)_ = 2.064, *p* = 0.1081; Figure 2A]. Group-housed vehicle control rats readily discriminated the objects during the choice trial, irrespective of subsequent acute injection of vehicle or lamotrigine, spending significantly more time (*p <* 0.001) exploring the novel than the familiar object, while isolated rats given vehicle (PCP-SI-V) or high dose lamotrigine (PCP-SI-L15) did not (*p* > 0.05). Of note, isolated rats receiving lamotrigine at 10mg/kg (PCP-SI-L10) spent significantly longer exploring the novel object (*p* < 0.001), showing that this dose reversed the NOR impairment induced by PCP-isolation-rearing.

(B) The D2 discrimination ratio (novel/novel + familiar times, mean ± SEM, n = 7–8) was significantly affected by PCP-SI-rearing [F_(1,31)_ = 25.731, *p* < 0.001] and lamotrigine [F_(2,30)_ = 9.795, *p* < 0.001], such that Bonferroni’s post hoc test showed a significant impairment in PCP-SI-V rats, **p < 0.01 from V-GH-V by was reversed by 10mg/kg lamotrigine, #*p* < 0.01 from PCP-SI-V following ANOVA.

Consistent with the raw data, there was a highly significant main effect of rearing condition [F_(1,31)_ = 25.731, *p* < 0.001] and drug treatment [F_(2,30)_ = 9.795, *p* < 0.001] but no interaction between the two [F_(1,31)_ = 0.844, *p* = 0.365] on the D2 discrimination ratio. Thus the D2 ratio was significantly lower in PCP-SI-V than V-GH-V (*p* < 0.01), and 10mg/kg lamotrigine rats (PCP-SI-L10) had a significantly higher D2 ratio than PCP-SI-V rats (*p* < 0.01; Figure 2B).

Consistent with interpretation that the effect of isolation and lamotrigine on NOR were specifically due to changes in learning and memory, no object preference was observed during the familiarization trial, and no change in total object exploration occurred between any groups during the choice trial (data not shown). Thus both isolation and lamotrigine caused a redistribution of exploration towards the unfamiliar object and not a non-specific reduction in overall object investigation in the choice trial.

### Effect of Lamotrigine on PCP-SI-Induced Attenuation of Prepulse Inhibition of Acoustic Startle

**Figure 3.**
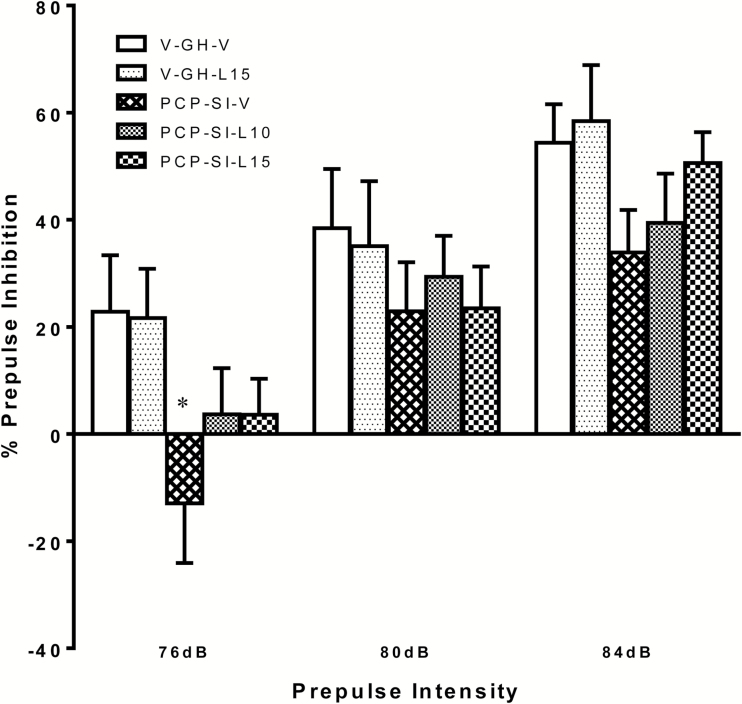
Impairment in prepulse inhibition (PPI) of acoustic startle response (at 76–84 dB prepulse intensity as indicated) by PCP-SI rearing was not reversed by lamotrigine treatment. PCP-SI reared rats (PCP-SI-V) demonstrated a significant impairment in PPI response (mean ± SEM, n = 7–8) compared to group-housed vehicle-treated littermates [V-GH-V; F_(1,32)_ = 4.447, *p* = 0.043, RM ANOVA]. The reduction in startle response was not reversed by lamotrigine treatment at either 10 (PCP-SI-L10) or 15mg/kg (PCP-SI-L15), and there were no between-factor interactions. *p < 0.05 from V-GH-V, Bonferroni’s post hoc test following ANOVA. PCP-SI, phencyclidine-treated pups housed alone in social isolation.

As expected, inhibition of the acoustic startle progressively increased with prepulse intensity irrespective of neurodevelopment condition or drug treatment. However, %PPI was lower in PCP-SI-V rats than in any other group at all prepulse intensities, such that ANOVA showed a significant main effect of prepulse [F_(2,64)_ = 105.060, *p* < 0.001] and rearing condition [F_(1,32)_ = 4.447, *p* = 0.043]. However, PCP-SI-rearing only significantly reduced %PPI at the 76 dB prepulse level (V-GH-V vs. PCP-SI-V, *p* < 0.05). The trend for %PPI to be increased by lamotrigine in the PCP-SI-L10 compared to the PCP-SI-V controls, most notably at 84 dB prepulse intensity, was not significant [F_(2,33)_ = 0.284, *p* = 0.754], and there was no rearing x lamotrigine interaction [F_(1,32)_ = 0.437, *p* = 0.513; Figure 3].

There was no significant effect of either rearing condition or lamotrigine, nor any interaction on the initial startle amplitude nor habituation to the 120 dB startle alone, confirming that observed effects were not confounded by any non-specific drug effect on the startle response (data not shown).

### PCP-SI-Induced Changes in Gene Expression

Microarray gene-expression profiling in the whole hippocampus (selected because of inferred relevance of this area to the neurobiological aetiology of schizophrenia and known neurochemical changes with isolation rearing in the rat) identified a total of 715 genes that were differentially expressed by the criteria defined in the methods. From these genes, only two were up-regulated, while 713 were down-regulated (Supplementary Table S1 and Table 2). A pathway analysis of the microarray gene-expression data identified the most likely biological systems and pathways perturbed by combined PCP and isolation. Of particular note, the most significantly affected pathways included glutamate metabolism and GABA and dopamine receptor signaling. Importantly, within each of these canonical pathways were genes either encoding proteins involved in rate limiting steps (e.g. glutamate decarboxylase and dopa decarboxylase) or transporters (GABA transporter) or receptors (GABA_B_) that have genetic associations as risk factors for schizophrenia (Harrison, 2015). In addition, IPA analysis indicated that PCP-SI altered several additional genes coding proteins (e.g. parvalbumin, neurotensin, and the 5-HT_5A_ receptor) previously associated by multiple techniques to changes seen in patients with schizophrenia (Beasley and Reynolds, 1997; Sharma et al., 1997; Iwata et al., 2001; Hashimoto et al., 2003; Table 2). The microarray data (Supplementary Table S1) also identified four gamma subunits of the L-type voltage-gated calcium channels to be down-regulated in PCP-SI rats, which will be discussed later. Of note, 34 genes encoding olfactory receptors and 3 encoding vomeronasal receptors (thought to respond to pheromones) were also down-regulated, but these changes may relate to the impact of social isolation on olfactory processes and have no relevance to changes in phenotype relevant to schizophrenia.

**Table 2.** Selection of Genes of Interest

Gene Symbol	Gene Assignment	*p*-value	q-value (%)	Fold Change
**Glutamate Metabolism Pathway**				
Gad1	glutamate decarboxylase 1	0.0284	47.72	-1.08
Gpt2	glutamic pyruvate transaminase (alanine aminotransferase) 2	0.0225	43.10	-1.08
Nadsyn1	NAD synthetase 1	0.0396	50.52	-1.08
Abat	4-aminobutyrate aminotransferase	0.0041	25.15	-1.06
Aldh5a1	aldehyde dehydrogenase 5 family, member A1	0.0239	43.10	-1.06
Glud1	glutamate dehydrogenase 1	0.0143	37.73	-1.05
**GABA Receptor Signaling Pathway**				
Gabra4	gamma-aminobutyric acid (GABA) A receptor, alpha 4	0.0085	32.49	-1.08
Gabrb2	gamma-aminobutyric acid (GABA) A receptor, beta 2	0.0065	27.96	-1.08
Slc6a1	solute carrier family 6 (neurotransmitter transporter, GABA), member 1	0.0291	47.72	-1.04
Gabbr1	gamma-aminobutyric acid (GABA) B receptor 1	0.0265	47.72	-1.03
**Also in pathway: Abat, Aldh5a1, Gad1 (see above**)				
**Dopamine Receptor Signaling Pathway**				
Ddc	dopa decarboxylase (aromatic L-amino acid decarboxylase)	0.0150	37.73	-1.24
Drd5	dopamine receptor D5	0.0265	47.72	-1.13
Gnal	guanine nucleotide binding protein, alpha activating activity polypeptide, olfactory type	0.0253	43.10	-1.13
Adcy1	adenylate cyclase 1 (brain)	0.0303	47.72	-1.10
Prkar2a	protein kinase, cAMP dependent regulatory, type II alpha	0.0209	43.10	-1.07
Ppp2r3a	protein phosphatase 2 (formerly 2A), regulatory subunit	0.0482	54.56	-1.06
Adcy2	adenylate cyclase 2 (brain)	0.0036	25.15	-1.06
Prkar1b	protein kinase, cAMP dependent regulatory, type I, beta	0.0433	50.52	-1.04
**Other Down-Regulated Genes of Interest**				
Nts	neurotensin	0.0064	27.96	-1.44
Npsr1	neuropeptide S receptor 1	0.0022	23.21	-1.18
Htr5a	5-hydroxytryptamine (serotonin) receptor 5A	0.0186	40.47	-1.11
Pvalb	parvalbumin	0.0048	25.15	-1.10
**Up-Regulated Genes**				
Csrp2	cysteine and glycine-rich protein 2	0.0001	9.75	1.15
Olr1459	olfactory receptor 1459	0.0001	9.75	1.11

Genes were selected based on differential expression in the hippocampus of PCP-SI rats compared to vehicle treated group-housed littermate controls by microarray analysis, following the selection criteria *p* < 0.05, q-val < 55%

From the microarray results, seven candidate differentially expressed genes (DEGs) of interest were selected for Q-PCR analysis which were from each of the three canonical pathways affected by PCP-SI. The DEGs were: GAD1 (glutamate metabolism to GABA); GABBR1, GABRA4, GABRB2, and SLC6A1 (GABA receptor signaling); and DRD5 and DDC (dopamine receptor signaling). These particular genes were selected because of their known association with schizophrenia (Allen et al., 2008; Ripke et al., 2013) and identification of their relative expression change from the current rat microarray data. Furthermore, Q-PCR was used to identify any change in expression of PVALB, previously known to be altered in the post-mortem brains of schizophrenia patients (Hardingham and Do, 2016), and by isolation rearing in the rat (Harte et al., 2007). Q-PCR analysis of these seven genes failed to show any significant alterations in gene expression when normalized to three housekeeping genes whose expression was unchanged in control and PCP-SI hippocampi (data not shown).

## Discussion

Overall, this study offers a new insight into the behavioral consequences and associated genetic background to complex neurodevelopmental alterations induced by combined neonatal PCP administration and isolation rearing of the rat, using animals paired by litter to minimize the impact of natural genetic variation. The results of the microarray suggest significant alterations in gene expression occur in three key neurotransmitter pathways believed to have relevance to the aetiology of schizophrenia. The implication that dopaminergic and GABAergic signaling, as well as glutamate metabolism, may be involved in the behavioral outcomes of combined PCP-SI treatment is similar to numerous findings in humans (Hashimoto et al., 2003; Benes et al., 2007; Howes and Kapur, 2009; Howes et al., 2012) and suggests the utility of this neurodevelopmental model to investigate the neurobiology of schizophrenia.

A large meta-analysis (Goff, 2009; Tiihonen et al., 2009) identified the potential benefit of lamotrigine to treat psychosis and schizoaffective disorder (Erfurth et al., 1998), in particular in clozapine-resistant patients, although other studies have failed to replicate this effect (Reid et al., 2013; Vayisoglu et al., 2013). In this study, lamotrigine significantly reduced hyperactivity in PCP-SI rats placed in a novel arena, a potential index of positive symptoms of schizophrenia (Fone and Porkess, 2008; Fabricius et al., 2010), but had no effect in V-GH littermates. In previous studies, the novelty-induced hyperactivity seen in GluA1 AMPA subunit knockout mice was suppressed following a 28 day dietary administration of lamotrigine (Maksimovic et al., 2014). Furthermore, Williams and colleagues (2006) showed combined administration of clozapine with lamotrigine significantly reduced acute PCP-induced hyperlocomotion. This data is consistent with clinical studies showing the benefit of combined clozapine and lamotrigine treatment on positive and negative symptoms in schizophrenia (Tiihonen et al., 2009). Lamotrigine reduces excitability in striatal neurons *in vitro* (Calabresi et al., 1999) by blockade of pre-synaptic voltage-gated sodium channels which attenuate neurotransmitter release (Leach et al., 1991). A similar attenuation of mesolimbic dopamine release could explain its ability to reduce hyperactivity in PCP-SI-rats and the psychosis in schizophrenia patients (Abi-Dargham et al., 2000), both thought to result from overactivity of the mesolimbic pathway. A recent study showed lamotrigine modulates phosphorylation of and disrupts the same βarr2/Akt/GSK3 signaling complex that is coupled to D_2_ dopamine receptors and was unable to suppress novelty-induced hyperactivity in βarr2-KO mice (Del’ Guidice and Beaulieu, 2015). This suggests lamotrigine may directly interfere with dopamine D_2_-mediated signaling *in vivo* but also that it might have limited use as an adjunct therapy with existing antipsychotics whose therapeutic effect on positive symptoms is likely to involve the D_2_ receptor antagonism.

Both isolation-rearing (Jones et al., 2011; Watson et al., 2012; McIntosh et al., 2013) and repeated PCP-injection to adult rats (McKibben et al., 2010; Grayson et al., 2015) impair NOR, as does combined PCP-SI (Gaskin et al., 2014; Watson et al., 2016), consistent with findings in this study. Lamotrigine at the low (10mg/kg i.p.) but not high dose restored the PCP-SI-induced impairment in NOR in this study. Few studies have examined the effect of lamotrigine on cognition, but in mice at 40mg/kg i.p. it prolonged the time in the learned quadrant in a Morris Water Maze probe trial of retention and improved latency in a passive avoidance test (Celikyurt et al., 2012). In contrast, lamotrigine did not cause any significant change in working memory performance (Shannon and Love, 2004) or attention in a five-choice serial reaction time task in rats (Shannon and Love, 2005).

Interestingly, the impairment in PPI in mGluR1 knock-out mice (Brody et al., 2003a) and methamphetamine-induced PPI deficits (Nakato et al., 2010) were both reversed by acute lamotrigine treatment, although amphetamine-induced deficits were not (Brody et al., 2003b). The current results don’t replicate these reports since the PCP-SI-induced deficit in PPI was unaffected by lamotrigine; however, previous publications used higher doses (27–30mg/kg) than herein. The current data are also consistent with our earlier findings (Gaskin et al., 2014) that PPI deficits induced by this dual-hit (neonatal PCP and isolation) treatment are more substantial than seen with isolation alone.

Previous microarray analysis of the prefrontal cortex 24h after perinatal PCP identified alterations in expression of genes involved in cell survival and apoptosis (Liu et al., 2010). PCP caused acute changes in brain derived neurotrophic factor (BDNF) and NMDAR1, akin to prefrontal changes seen in schizophrenia patients and some rodent models for the disorder (Beasley and Reynolds, 1997; Liu et al., 2010). Previous microarray studies found altered expression of the NR2A NMDA receptor subunit and development-related genes (e.g. NURR1) in the prefrontal cortex (Turnock-Jones et al., 2009) and the dentate gyrus (Ibi et al., 2008) of isolation-reared rodents. However, this is the first study to report long-term effects of neonatal PCP (and isolation rearing) on gene expression.

Ingenuity Pathway Analysis herein clearly identified alterations in three canonical pathways, which are highly relevant to changes thought to occur in schizophrenia. A central tenet of schizophrenia aetiology is alteration in dopaminergic pathology in mesolimbic and mesocortical pathways (Howes and Kapur, 2009; Howes et al., 2012), so the observation that dopamine signaling was significantly affected by PCP-SI is not surprising. Dopamine projections to the hippocampus are sparse but thought to regulate ventral cholinergic projections to the nucleus accumbens (nAcc; Floresco et al., 2001) and a feedback loop from the ventral tegmental area to the ventral hippocampus is implicated in object-based memory formation through long-term potentiation and long-term depression. The current microarray finding that the DRD5 gene encoding the dopamine D_5_ receptor was down-regulated, together with two variants of adenylate cyclase, the down-stream effector of this G-protein receptor, is particularly interesting. The 1.24-fold decrease in the DDC gene reported herein in PCP-SI rats is also unsurprising. Polymorphisms in DDC, the gene the encoding dopa decarboxylase which converts L-DOPA into dopamine, have been associated with the age of onset of schizophrenia in male patients (Borglum et al., 2001), although other studies found no association in paranoid schizophrenia (Hoogendoorn et al., 2005; Talkowski et al., 2008).

The role of glutamate in schizophrenia has been widely studied and is thought to contribute to the negative and cognitive deficits. In the current study, glutamate signaling was relatively unaffected by PCP-SI, with none of the NMDA receptor subunits being differentially expressed, although genes coding proteins involved in glutamate metabolism were significantly affected. Specifically, six genes encoding enzymes regulating glutamate metabolism were significantly down-regulated in PCP-SI rats, as well as one gene encoding the transporter protein, SLC1A3 (Solute carrier family 1 member 3, aka Glutamate Aspartate Transporter or Excitatory Amino Acid Transporter 1; Table 2). It is unclear if down-regulation of these genes is a compensatory result of lower basal glutamate in PCP-SI rats or a direct consequence of the developmental manipulation. Of particular note, GAD67 (aka Glutamate Decarboxylase 1, 67kDa isoform), encoding the enzyme which converts glutamate to GABA, is down-regulated in both the hippocampi (Benes et al., 2007) and cortices (Hashimoto et al., 2003) of schizophrenia patients and the medial pre-frontal cortices of rats injected with MK-801 on PND 7 and socially isolated post-weaning (Gilabert-Juan et al., 2012). Similarly, ketamine application to cultured hippocampal neurons caused a dose-dependent decrease in GAD67 immunoreactivity in parvalbumin-expressing GABAergic interneurons (Kinney et al., 2006), but this is the first study to suggest similar changes occur following neonatal PCP.

**Figure 4.**
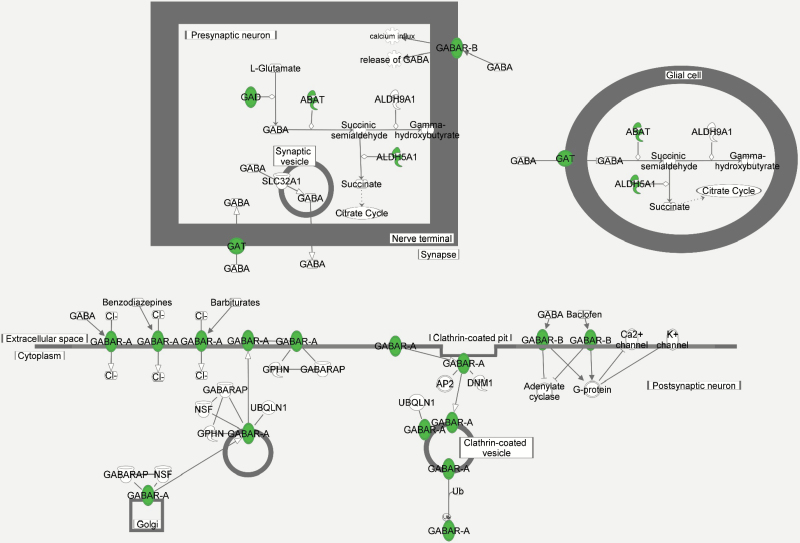
Pictorial representation of altered GABA receptor signaling due to PCP-SI treatment. The proteins highlighted (filled circle over-written with name as text) correspond with mRNA levels found to be differentially expressed in PCP-SI rats following microarray analysis, all of which were identified as being down-regulated (see Table 2). Figure generated using Ingenuity Pathway Analysis (IPA, Ingenuity Systems Inc., QIAGEN). PCP-SI, phencyclidine-treated pups housed alone in social isolation.

Interestingly, neonatal PCP alone decreases the sensitivity to a GAT1 inhibitor (Kjaerby et al., 2014), consistent with the microarray data herein showing decreased expression of the GABA transporter gene SLC6A1 (Table 2). A variety of GABA-associated genes were also down-regulated by PCP-SI treatment, including the PVALB gene encoding parvalbumin and three GABA receptor subunit genes (GABBR1, GABRA4, GABRB2; Table 2, Figure 4). Impaired GABAergic inhibition of hippocampal neural activity is emerging as a key feature of schizophrenia (Lisman et al., 2008; Heckers and Konradi, 2010). Patients have altered post-mortem levels of markers of GABA neurone integrity and parvalbumin (Beasley and Reynolds, 1997; Heckers and Konradi, 2010). Imaging studies also show hippocampal metabolic hyperactivity (Heckers et al., 1998; Tamminga et al., 2010; Schobel et al., 2013; Tregellas et al., 2014) in patients with schizophrenia, which may contribute to excitotoxic volume reduction (Adriano et al., 2012). Consistent with these human findings, isolation-reared rats have loss of parvalbumin-positive GABAergic neurones (Harte et al., 2007) and reduction in markers of dendrites, synapses (Varty et al., 1999), and microtubular proteins (Bianchi et al., 2006), together with impaired GABA release, measured using magnetic resonance spectroscopy (Napolitano et al., 2013), which may cause decreased hippocampal plasticity and function. Q-PCR analysis failed to confirm the down-regulation of genes identified by canonical pathway analysis, probably because the small fold-changes were below detection levels. As loss of parvalbumin-positive interneurons is localized to the CA1 and CA2/3 hippocampal regions and not seen in the dentate gyrus of sub-chronic PCP-treated rats (Jenkins et al., 2010), measurement in the whole hippocampus used herein may have prevented detection of change by Q-PCR. Nonetheless, the data support the concept that facilitation of GABAergic activity may be a mechanism to develop future adjunctive therapeutics for schizophrenia (Lodge and Grace, 2011), which was evaluated in the current study by attempting to reverse behavioral deficits of PCP-SI rats using acute injection of lamotrigine. Increasing evidence from human genetic associations implies that genes implicated in schizophrenia show convergence on common biological pathways, such as the NMDA receptor signaling complex and voltage-gated calcium channels associated with the post-synaptic density at glutamatergic synapsis involved in neuronal plasticity (Hall et al., 2015). Of note, the current microarray data (Supplementary Table S1) identified four gamma subunits of the L-type voltage-gated calcium channels to be down-regulated in PCP-SI rats, and single nucleotide polymorphisms in two of these (CACNG4 and CACNG6) have recently also been associated with schizophrenia (Guan et al., 2016). Conversely, many genes previously associated with schizophrenia do not appear in the list identified herein. This is not surprising since meta-analysis and genome-wide association studies show that thousands of common single nucleotide polymorphisms exist, but each has a small effect (odds ratios 1.1 to 1.2), only cumulatively explaining about 30% of the underlying genetic risk of schizophrenia (Allen et al., 2008; Ripke et al., 2013). Given the level of these associations, it would be surprising if the current analysis had sufficient power to identify individual genes, and instead has identified likely pathways affected.

In conclusion, neonatal PCP treatment combined with subsequent isolation rearing of rats induces changes in expression of hippocampal genes regulating dopamine and GABA signaling and glutamate metabolism, several of which involve proteins strongly associated with schizophrenia. Acute lamotrigine treatment partially reversed locomotor hyperactivity and (at the low dose) NOR impairment caused by PCP-SI. The relevance of these changes to the positive and cognitive symptoms of schizophrenia suggests that the therapeutic potential of drugs targeting voltage-gated sodium channels and modulating GABAergic and glutamatergic pathways as adjunctive therapy for schizophrenia is warranted.

## Statement of Interest

None.

## Supplementary Material

Supplementary Table S1
