# Supplementary material for: Down-Regulation of Hippocampal Genes Regulating Dopaminergic, GABAergic, and Glutamatergic Function Following Combined Neonatal Phencyclidine and Post-Weaning Social Isolation of Rats as a Neurodevelopmental Model for Schizophrenia
Source: Int J Neuropsychopharmacol. 2016 Jul 5;19(11):pyw062. doi: 10.1093/ijnp/pyw062 (PMC5137279; doi:10.1093/ijnp/pyw062)
Supplement: Supplementary Table S1 [file Supplementary_Table_S1.docx]

Supplementary Table S1

Genes identified as significantly up- or down-regulated in the hippocampus of adult rats following combined neonatal phencyclidine treatment and post-weaning social isolation compared to saline treated group-housed littermates. Eight litters of three-day old, male Lister hooded rat pups accompanied by their natural dam received On phencyclidine hydrochloride (PCP, 10mg/kg s.c., n=8) on PND 7, 9 and 11 or saline (1ml/kg, n=8). Pups were weaned by litter on PND23, those receiving saline were housed in groups of 3-4 while PCP treated pups were housed alone in social isolation for the rest of the study. Rats were examined for activity in an open field but had no further behavioural testing and on PND 70 rats were killed by schedule 1 and the hippocampus rapidly removed and frozen at -80^O^C for later anaylsis. RNA samples were hybridized onto Affymetrix GeneChip Rat Exon Gene 1.0 ST microarrays (Affymetrix, Santa Clara, CA, USA) and analysed to determine change in expression meeting lenient critical thresholds of a combined change in expression (*p*<0.05) and q-value < 55%. Genes meeting criteria were entered into the Ingenuity Pathway Analysis software (IPA, Ingenuity Systems Inc., QIAGEN, Redwood City, CA., USA) from which the table below was obtained.

| Gene Symbol | *Gene Assignment* | Ref Seq | P value | q-value(%) | Fold Change |
| --- | --- | --- | --- | --- | --- |
| Nts | *neurotensin* | NM_001102381 | 0.0064 | 27.96 | -1.44 |
| Cd3e | *CD3 molecule, epsilon polypeptide* | NM_001108140 | 0.0057 | 27.96 | -1.32 |
| RGD1311318 | *similar to Arylacetamide deacetylase (AADAC)* | XM_001062078 | 0.0004 | 12.18 | -1.31 |
| C1ql2 | *complement component 1, q subcomponent-like 2* | NM_001105949 | 0.0002 | 0.00 | -1.28 |
| Olr1326 | *olfactory receptor 1326* | NM_001000474 | 0.0229 | 43.10 | -1.26 |
| Scgn | *secretagogin, EF-hand calcium binding protein* | NM_201561 | 0.0003 | 9.75 | -1.25 |
| Ndst4 | *N-deacetylase* | NM_001191849 | 0.0035 | 25.15 | -1.25 |
| Olr1323 | *olfactory receptor 1323* | NM_001000472 | 0.0019 | 23.21 | -1.25 |
| Olr334 | *olfactory receptor 334* | NM_001001041 | 0.0090 | 32.49 | -1.24 |
| Ddc | *dopa decarboxylase (aromatic L-amino acid decarboxylase)* | NM_012545 | 0.0150 | 37.73 | -1.24 |
| Akr1c12 | *aldo-keto reductase family 1, member C12* | NM_001170342 | 0.0073 | 29.67 | -1.22 |
| Olr311 | *olfactory receptor 311* | NM_001000555 | 0.0118 | 35.28 | -1.21 |
| LOC100361732 | *ribosomal protein S19-like* | ENSRNOT00000037731 | 0.0490 | 54.56 | -1.21 |
| RGD1307396 | *similar to RIKEN cDNA 6330406I15* | ENSRNOT00000001205 | 0.0022 | 23.21 | -1.20 |
| Olr576 | *olfactory receptor 576* | NM_001000328 | 0.0358 | 50.52 | -1.19 |
| Olr1085 | *olfactory receptor 1085* | NM_001000420 | 0.0112 | 35.28 | -1.19 |
| Olr1068 | *olfactory receptor 1068* | NM_001001077 | 0.0244 | 43.10 | -1.19 |
| Prox1 | *prospero homeobox 1* | NM_001107201 | 0.0004 | 9.75 | -1.18 |
| Tas2r110 | *taste receptor, type 2, member 110* | NM_001166677 | 0.0205 | 43.10 | -1.18 |
| Npsr1 | *neuropeptide S receptor 1* | ENSRNOT00000021625 | 0.0022 | 23.21 | -1.18 |
| Gjc3 | *gap junction protein, gamma 3* | ENSRNOT00000001797 | 0.0125 | 35.28 | -1.18 |
| Olr1241 | *olfactory receptor 1241* | NM_001000449 | 0.0185 | 40.47 | -1.18 |
| Tmem144 | *transmembrane protein 144* | NM_001108551 | 0.0088 | 32.49 | -1.17 |
| Sv2c | *synaptic vesicle glycoprotein 2c* | NM_031593 | 0.0129 | 35.28 | -1.17 |
| Olr404 | *olfactory receptor 404* | NM_001000382 | 0.0497 | 54.56 | -1.17 |
| Ubl4 | *ubiquitin-like 4* | NM_001106346 | 0.0018 | 23.21 | -1.17 |
| Ubl4 | *ubiquitin-like 4* | NM_001106346 | 0.0034 | 25.15 | -1.17 |
| Olr227 | *olfactory receptor 227* | NM_001000203 | 0.0100 | 35.28 | -1.17 |
| Olr670 | *olfactory receptor 670* | NM_001000633 | 0.0336 | 47.72 | -1.17 |
| Olr1223 | *olfactory receptor 1223* | NM_001000441 | 0.0135 | 37.73 | -1.17 |
| Gjc2 | *gap junction protein, gamma 2* | NM_001100784 | 0.0148 | 37.73 | -1.16 |
| Vom2r31 | *vomeronasal 2 receptor, 31* | NM_173319 | 0.0089 | 32.49 | -1.16 |
| Lrrc10b | *leucine rich repeat containing 10B* | NM_001107577 | 0.0338 | 47.72 | -1.16 |
| C1ql3 | *complement component 1, q subcomponent-like 3* | NM_001109403 | 0.0002 | 9.75 | -1.16 |
| Scn7a | *sodium channel, voltage-gated, type VII, alpha* | ENSRNOT00000040299 | 0.0472 | 54.56 | -1.16 |
| Mir133b | *microRNA mir-133b* | NR_032117 | 0.0001 | 0.00 | -1.16 |
| Il23r | *interleukin 23 receptor* | XM_001072576 | 0.0310 | 47.72 | -1.16 |
| Olr183 | *olfactory receptor 183* | NM_001000181 | 0.0482 | 54.56 | -1.16 |
| Sass6 | *spindle assembly 6 homolog (C. elegans)* | NM_001107719 | 0.0138 | 37.73 | -1.16 |
| Slmo1 | *slowmo homolog 1 (Drosophila)* | NM_001109570 | 0.0006 | 12.18 | -1.16 |
| Olr728 | *olfactory receptor 728* | NM_001000363 | 0.0082 | 32.49 | -1.16 |
| Arg1 | *arginase, liver* | NM_017134 | 0.0145 | 37.73 | -1.16 |
| RGD1306186 | *similar to RIKEN cDNA 4930569K13* | BC090317 | 0.0490 | 54.56 | -1.16 |
| LOC689770 | *similar to osteoclast inhibitory lectin* | NM_001142304 | 0.0269 | 47.72 | -1.16 |
| Olr1137 | *olfactory receptor 1137* | NM_001001079 | 0.0304 | 47.72 | -1.15 |
| Olr1166 | *olfactory receptor 1166* | NM_001000867 | 0.0347 | 50.52 | -1.15 |
| Ntn1 | *netrin 1* | AY028417 | 0.0242 | 43.10 | -1.15 |
| Capn6 | *calpain 6* | NM_031808 | 0.0162 | 40.47 | -1.15 |
| Diras2 | *DIRAS family, GTP-binding RAS-like 2* | NM_001169578 | 0.0016 | 20.52 | -1.15 |
| Olr774 | *olfactory receptor 774* | NM_001000918 | 0.0360 | 50.52 | -1.15 |
| Tal2 | *T-cell acute lymphocytic leukemia 2* | NM_001109462 | 0.0242 | 43.10 | -1.15 |
| Rwdd2b | *RWD domain containing 2B* | NM_001100559 | 0.0035 | 25.15 | -1.15 |
| Galntl6 | *N-Acetylgalactosaminyltransferase-Like 6* | NM_001135756 | 0.0001 | 0.00 | -1.15 |
| Senp18 | *Sumo1* | NM_001002834 | 0.0042 | 25.15 | -1.15 |
| Olr1016 | *olfactory receptor 1016* | NM_001000070 | 0.0212 | 43.10 | -1.14 |
| Cd274 | *CD274 molecule* | NM_001191954 | 0.0104 | 35.28 | -1.14 |
| Egln1 | *EGL nine homolog 1 (C. elegans)* | AY228140 | 0.0096 | 32.49 | -1.14 |
| LOC100361629 | *ERBB2 interacting protein-like* | ENSRNOT00000061017 | 0.0005 | 12.18 | -1.14 |
| LOC691196 | *similar to arylacetamide deacetylase* | ENSRNOT00000044701 | 0.0024 | 23.21 | -1.14 |
| Mycl1 | *v-myc myelocytomatosis viral oncogene homolog 1, lung c* | NM_001191763 | 0.0027 | 23.21 | -1.14 |
| Grasp | *GRP1 (general receptor for phosphoinositides 1)-associated* | NM_138894 | 0.0074 | 29.67 | -1.14 |
| Fen1 | *flap structure-specific endonuclease 1* | NM_053430 | 0.0006 | 12.18 | -1.14 |
| Defb2 | *defensin beta 2* | NM_001037507 | 0.0468 | 54.56 | -1.14 |
| RGD1563891 | *similar to core 2 beta-1,6-N-acetylglucosamine* | ENSRNOT00000052189 | 0.0074 | 29.67 | -1.14 |
| RGD1306622 | *similar to KIAA0954 protein* | NM_001170487 | 0.0093 | 32.49 | -1.14 |
| Scn4b | *sodium channel, voltage-gated, type IV, beta* | NM_001008880 | 0.0276 | 47.72 | -1.14 |
| Vom2r59 | *vomeronasal 2 receptor, 59* | NM_001099466 | 0.0315 | 47.72 | -1.14 |
| Hbegf | *heparin-binding EGF-like growth factor* | NM_012945 | 0.0030 | 25.15 | -1.14 |
| Vom1r96 | *vomeronasal 1 receptor, 96* | NM_001008927 | 0.0037 | 25.15 | -1.14 |
| Olr279 | *olfactory receptor 279* | NM_001000226 | 0.0477 | 54.56 | -1.14 |
| Zfp563 | *zinc finger protein 563* | NM_001134561 | 0.0047 | 25.15 | -1.14 |
| Sike | *suppressor of IKK epsilon* | NM_001012182 | 0.0009 | 15.00 | -1.14 |
| Sertad4 | *SERTA domain containing 4* | NM_001108351 | 0.0012 | 17.72 | -1.13 |
| Depdc7 | *DEP domain containing 7* | NM_001029916 | 0.0460 | 54.56 | -1.13 |
| Itpka | *inositol 1,4,5-trisphosphate 3-kinase A* | NM_031045 | 0.0045 | 25.15 | -1.13 |
| LOC100361960 | *rCG57154-like* | ENSRNOT00000059499 | 0.0186 | 40.47 | -1.13 |
| H1fnt | *H1 histone family, member N, testis-specific* | NM_001024356 | 0.0448 | 50.52 | -1.13 |
| Sim1 | *single-minded homolog 1 (Drosophila)* | NM_001107641 | 0.0001 | 0.00 | -1.13 |
| Olr483 | *olfactory receptor 483* | NM_001000683 | 0.0257 | 43.10 | -1.13 |
| Ier5 | *immediate early response 5* | NM_001025137 | 0.0346 | 47.72 | -1.13 |
| Api5 | *apoptosis inhibitor 5* | NM_001127379 | 0.0006 | 12.18 | -1.13 |
| Trim59 | *tripartite motif-containing 59* | NM_001108945 | 0.0458 | 54.56 | -1.13 |
| Armcx3 | *armadillo repeat containing, X-linked 3* | NM_001014273 | 0.0005 | 12.18 | -1.13 |
| Olr1617 | *olfactory receptor 1617* | NM_001000839 | 0.0140 | 37.73 | -1.13 |
| Olr19 | *olfactory receptor 19* | NM_001000117 | 0.0392 | 50.52 | -1.13 |
| Drd5 | *dopamine receptor D5* | NM_012768 | 0.0265 | 47.72 | -1.13 |
| Pdcd4 | *programmed cell death 4* | NM_022265 | 0.0002 | 0.00 | -1.13 |
| RGD1565672 | *similar to MDM2 Binding protein* | NM_001130717 | 0.0372 | 50.52 | -1.13 |
| Gpr37 | *G protein-coupled receptor 37* | NM_057201 | 0.0103 | 35.28 | -1.13 |
| Gnal | *guanine nucleotide binding protein, alpha stimulating, olfactory type* | NM_001191836 | 0.0253 | 43.10 | -1.13 |
| LOC679989 | *similar to t-complex 11 protein* | ENSRNOT00000057153 | 0.0090 | 32.49 | -1.13 |
| Olr1294 | *olfactory receptor 1294* | NM_001000597 | 0.0445 | 50.52 | -1.13 |
| Rap1a | *RAP1A, member of RAS oncogene family* | NM_001005765 | 0.0059 | 27.96 | -1.13 |
| Btg2 | *BTG family, member 2* | NM_017259 | 0.0036 | 25.15 | -1.13 |
| Rad9b | *RAD9 homolog B (S. cerevisiae)* | NM_001030042 | 0.0039 | 25.15 | -1.13 |
| Olr1462 | *olfactory receptor 1462* | NM_001001097 | 0.0388 | 50.52 | -1.13 |
| Glb1l3 | *galactosidase, beta 1-like 3* | NM_001024358 | 0.0302 | 47.72 | -1.13 |
| Ndrg1 | *N-myc downstream regulated gene 1* | NM_001011991 | 0.0123 | 35.28 | -1.13 |
| Rprml | *reprimo-like* | NM_001109481 | 0.0107 | 35.28 | -1.12 |
| Olr720 | *olfactory receptor 720* | NM_001001067 | 0.0271 | 47.72 | -1.12 |
| Hapln4 | *hyaluronan and proteoglycan link protein 4* | NM_001108398 | 0.0129 | 35.28 | -1.12 |
| Zg16 | *zymogen granule protein 16* | NM_134409 | 0.0158 | 40.47 | -1.12 |
| Rpp14 | *ribonuclease P 14 subunit (human)* | NM_001108372 | 0.0122 | 35.28 | -1.12 |
| Ccne1 | *cyclin E1* | NM_001100821 | 0.0152 | 37.73 | -1.12 |
| Kcna6 | *potassium voltage gated channel, shaker related subfamily, member 6* | NM_023954 | 0.0050 | 25.15 | -1.12 |
| Srd5a2 | *steroid-5-alpha-reductase, alpha polypeptide 2 (3-oxo-5 a* *-Oxo-5 Alpha-Steroid Delta 4-Dehydrogenase Alpha 2)* | NM_022711 | 0.0311 | 47.72 | -1.12 |
| Pgbd5 | *piggyBac transposable element derived 5* | NM_001106198 | 0.0027 | 23.21 | -1.12 |
| Plscr1 | *phospholipid scramblase 1* | NM_057194 | 0.0037 | 25.15 | -1.12 |
| Mir433 | *microRNA mir-433* | NR_031954 | 0.0311 | 47.72 | -1.12 |
| Ghsr | *growth hormone secretagogue receptor* | NM_032075 | 0.0343 | 47.72 | -1.12 |
| Utp15 | *UTP15, U3 small nucleolar ribonucleoprotein, homolog (S Cerevisiae)* | NM_001107647 | 0.0032 | 25.15 | -1.12 |
| Ighg | *Immunoglobulin heavy chain (gamma polypeptide)* | BC092584 | 0.0475 | 54.56 | -1.12 |
| Olr1423 | *olfactory receptor 1423* | NM_001000006 | 0.0449 | 54.56 | -1.12 |
| Kcnk2 | *potassium channel, subfamily K, member 2* | NM_172042 | 0.0251 | 43.10 | -1.12 |
| Lingo3 | *leucine rich repeat and Ig domain containing 3* | NM_001109609 | 0.0436 | 50.52 | -1.12 |
| LOC680988 | *similar to ribosomal protein S12* | ENSRNOT00000052241 | 0.0209 | 43.10 | -1.12 |
| Lap3 | *leucine aminopeptidase 3* | NM_001011910 | 0.0061 | 27.96 | -1.12 |
| Stxbp6 | *syntaxin binding protein 6 (amisyn)* | NM_001191872 | 0.0010 | 17.72 | -1.12 |
| Pip4k2a | *phosphatidylinositol-5-phosphate 4-kinase, type II, alpha* | NM_053926 | 0.0007 | 12.18 | -1.12 |
| Olr1148 | *olfactory receptor 1148* | NM_001000875 | 0.0340 | 47.72 | -1.12 |
| Neto2 | *neuropilin (NRP) ad tolloid (TLL)-like 2* | NM_001107417 | 0.0117 | 35.28 | -1.12 |
| Rab33b | *RAB33B, member of RAS oncogene family* | NM_001108944 | 0.0004 | 12.18 | -1.12 |
| Gpc4 | *glypican 4* | NM_001014108 | 0.0026 | 23.21 | -1.12 |
| Serpina7 | *Serpin Peptidase Inhibitor, Clade A (Alpha-1 Antiproteinase, Antitrypsin), Member 7* | ENSRNOT00000014739 | 0.0465 | 54.56 | -1.12 |
| Ttll2 | *tubulin tyrosine ligase-like family, member 2* | ENSRNOT00000044831 | 0.0230 | 43.10 | -1.11 |
| Aldh3a2 | *aldehyde dehydrogenase 3 family, member A2* | NM_031731 | 0.0159 | 40.47 | -1.11 |
| Ppp1r16b | *protein phosphatase 1, regulatory (inhibitor) subunit 16b* | NM_001191072 | 0.0022 | 23.21 | -1.11 |
| Serpinb6b | *serine (or cysteine) peptidase inhibitor, clade B* | NM_001012214 | 0.0140 | 37.73 | -1.11 |
| Emilin2 | *elastin microfibril interfacer 2* | ENSRNOT00000056272 | 0.0441 | 50.52 | -1.11 |
| Nkx6-2 | *NK6 homeobox 2* | NM_001107558 | 0.0251 | 43.10 | -1.11 |
| Ablim3 | *actin-binding LIM protein 3* | NM_001191698 | 0.0061 | 27.96 | -1.11 |
| Unc84b | *unc-84 homolog B (C. elegans)* | ENSRNOT00000046399 | 0.0080 | 32.49 | -1.11 |
| Vangl2 | *vang-like 2 (van gogh, Drosophila)* | NM_001105969 | 0.0046 | 25.15 | -1.11 |
| Dgat2 | *diacylglycerol O-acyltransferase homolog 2 (mouse)* | NM_001012345 | 0.0046 | 25.15 | -1.11 |
| Rassf6 | *Ras Association (RalGDS/AF-6) Domain Family Member 6* | NM_001025671 | 0.0479 | 54.56 | -1.11 |
| Ctns | *cystinosis, nephropathic* | NM_001191647 | 0.0299 | 47.72 | -1.11 |
| Rph3a | *rabphilin 3A* | NM_133518 | 0.0014 | 20.07 | -1.11 |
| LOC100365853 | *zinc finger protein 623-like* | ENSRNOT00000012321 | 0.0337 | 47.72 | -1.11 |
| Aif1l | *allograft inflammatory factor 1-like* | NM_001108578 | 0.0204 | 43.10 | -1.11 |
| N-pac | *cytokine-like nuclear factor n-pac* | NM_001007800 | 0.0015 | 20.52 | -1.11 |
| LOC498029 | *similar to RIKEN cDNA A730011L01 gene* | BC088247 | 0.0006 | 12.18 | -1.11 |
| Cdh19 | *cadherin 19, type 2* | NM_001009448 | 0.0488 | 54.56 | -1.11 |
| Lrp1b | *Low Density Lipoprotein Receptor-Related Protein 1B* | NM_001107843 | 0.0153 | 37.73 | -1.11 |
| Htra1 | *HtrA serine peptidase 1* | NM_031721 | 0.0156 | 37.73 | -1.11 |
| Klk11 | *kallikrein related-peptidase 11* | NM_001106252 | 0.0411 | 50.52 | -1.11 |
| RGD1565767 | *similar to ribosomal protein L15* | ENSRNOT00000032528 | 0.0286 | 47.72 | -1.11 |
| Htr5a | *5-hydroxytryptamine (serotonin) receptor 5A* | NM_013148 | 0.0186 | 40.47 | -1.11 |
| LOC500164 | *rCG64160-like* | ENSRNOT00000047620 | 0.0300 | 47.72 | -1.11 |
| Lix1l | *Lix1 homolog (mouse)-like* | NM_001024303 | 0.0047 | 25.15 | -1.11 |
| Hace1 | *HECT domain and ankyrin repeat containing, E3 ubiquitin* | NM_001108539 | 0.0028 | 23.21 | -1.11 |
| Kcnh7 | *potassium voltage-gated channel, subfamily H (eag-related)* | NM_131912 | 0.0293 | 47.72 | -1.11 |
| Trnp1 | *TMF1-regulated nuclear protein 1* | ENSRNOT00000032841 | 0.0055 | 27.96 | -1.11 |
| Il1rap | *interleukin 1 receptor accessory protein* | NM_012968 | 0.0040 | 25.15 | -1.11 |
| Stk32b | *serine* | NM_001107224 | 0.0297 | 47.72 | -1.11 |
| Mars2 | *methionyl-tRNA synthetase 2, mitochondrial* | ENSRNOT00000021140 | 0.0138 | 37.73 | -1.11 |
| Pde7b | *phosphodiesterase 7B* | NM_080894 | 0.0006 | 12.18 | -1.11 |
| RGD1561195 | *similar to ribosomal protein L31* | ENSRNOT00000046725 | 0.0458 | 54.56 | -1.11 |
| Tdo2 | *tryptophan 2,3-dioxygenase* | NM_022403 | 0.0247 | 43.10 | -1.11 |
| Dclk2 | *doublecortin-like kinase 2* | NM_001195832 | 0.0320 | 47.72 | -1.11 |
| RGD1307595 | *similar to RIKEN cDNA 1700018B24* | NM_001134511 | 0.0012 | 17.72 | -1.10 |
| Klf13 | *Kruppel-like factor 13* | NM_001109147 | 0.0095 | 32.49 | -1.10 |
| Ermn | *ermin, ERM-like protein* | NM_001008311 | 0.0287 | 47.72 | -1.10 |
| Stox2 | *storkhead box 2* | NM_001134863 | 0.0012 | 17.72 | -1.10 |
| Cdh17 | *cadherin 17* | NM_053977 | 0.0044 | 25.15 | -1.10 |
| RGD1560277 | *similar to RIKEN cDNA D330012F22 gene* | XM_001066043 | 0.0060 | 27.96 | -1.10 |
| Mettl10 | *methyltransferase like 10* | NM_001108504 | 0.0004 | 9.75 | -1.10 |
| Olr1159 | *olfactory receptor 1159* | NM_001000871 | 0.0384 | 50.52 | -1.10 |
| S1pr5 | *sphingosine-1-phosphate receptor 5* | NM_021775 | 0.0300 | 47.72 | -1.10 |
| Polr3f | *polymerase (RNA) III (DNA directed) polypeptide F* | NM_001107784 | 0.0202 | 43.10 | -1.10 |
| Slc27a1 | *solute carrier family 27 (fatty acid transporter), member 1* | NM_053580 | 0.0423 | 50.52 | -1.10 |
| Prmt8 | *protein arginine methyltransferase 8* | ENSRNOT00000006878 | 0.0038 | 25.15 | -1.10 |
| Pla2g7 | *Phospholipase A2, Group VII (Platelet-Activating Factor Acetylhydrolase, Plasma)* | NM_001009353 | 0.0307 | 47.72 | -1.10 |
| Slc39a8 | *solute carrier family 39 (zinc transporter), member 8* | NM_001011952 | 0.0480 | 54.56 | -1.10 |
| Gatm | *glycine amidinotransferase (L-arginine:glycine amidinotrans* | NM_031031 | 0.0026 | 23.21 | -1.10 |
| Crnkl1 | *crooked neck pre-mRNA splicing factor-like 1 (Drosophila)* | NM_053797 | 0.0047 | 25.15 | -1.10 |
| Mixl1 | *Mix1 homeobox-like 1 (Xenopus laevis)* | NM_001105979 | 0.0168 | 40.47 | -1.10 |
| Kctd2 | *potassium channel tetramerisation domain containing 2* | ENSRNOT00000032608 | 0.0339 | 47.72 | -1.10 |
| Fgf2 | *fibroblast growth factor 2* | NM_019305 | 0.0087 | 32.49 | -1.10 |
| Olr1049 | *olfactory receptor 1049* | NM_001001365 | 0.0293 | 47.72 | -1.10 |
| LOC100364077 | *UTP14, U3 small nucleolar ribonucleoprotein* | ENSRNOT00000006672 | 0.0370 | 50.52 | -1.10 |
| Hyal6 | *hyaluronoglucosaminidase 6* | NM_001024320 | 0.0071 | 29.67 | -1.10 |
| Wscd1 | *WSC domain containing 1* | NM_001024234 | 0.0065 | 27.96 | -1.10 |
| Tmtc2 | *transmembrane and tetratricopeptide repeat containing 2* | XM_001080732 | 0.0438 | 50.52 | -1.10 |
| Cd47 | *Cd47 molecule* | NM_019195 | 0.0023 | 23.21 | -1.10 |
| Cdca4 | *cell division cycle associated 4* | NM_001037214 | 0.0248 | 43.10 | -1.10 |
| Spock3 | *sparc* | NM_001107310 | 0.0153 | 37.73 | -1.10 |
| Lppr1 | *lipid phosphate phosphatase-related protein type 1* | NM_201271 | 0.0237 | 43.10 | -1.10 |
| Eps8 | *epidermal growth factor receptor pathway substrate* | ENSRNOT00000009328 | 0.0089 | 32.49 | -1.10 |
| Dcaf5 | *DDB1 and CUL4 associated factor 5* | NM_001100718 | 0.0043 | 25.15 | -1.10 |
| RGD1560812 | *RGD1560812* | XM_576015 | 0.0049 | 25.15 | -1.10 |
| Gpc5 | *glypican 5* | NM_001107285 | 0.0168 | 40.47 | -1.10 |
| Sstr2 | *somatostatin receptor 2* | NM_019348 | 0.0495 | 54.56 | -1.10 |
| Nefh | *neurofilament, heavy polypeptide* | NM_012607 | 0.0114 | 35.28 | -1.10 |
| Zfp354c | *zinc finger protein 354C* | NM_023988 | 0.0048 | 25.15 | -1.10 |
| Fah | *fumarylacetoacetate hydrolase* | NM_017181 | 0.0438 | 50.52 | -1.10 |
| Adcy1 | *adenylate cyclase 1 (brain)* | NM_001107239 | 0.0303 | 47.72 | -1.10 |
| Rftn2 | *raftlin family member 2* | ENSRNOT00000020910 | 0.0364 | 50.52 | -1.10 |
| Pvalb | *parvalbumin* | NM_022499 | 0.0048 | 25.15 | -1.10 |
| Sall2 | *sal-like 2 (Drosophila)* | NM_001107262 | 0.0205 | 43.10 | -1.10 |
| RGD1565635 | *similar to zinc finger protein 124* | ENSRNOT00000021981 | 0.0266 | 47.72 | -1.10 |
| Ehd3 | *EH-domain containing 3* | NM_138890 | 0.0006 | 12.18 | -1.10 |
| Ugt2a1 | *UDP glucuronosyltransferase 2 family, polypeptide A1* | NM_022228 | 0.0136 | 37.73 | -1.10 |
| Gpr31 | *G protein-coupled receptor 31* | NM_001169132 | 0.0394 | 50.52 | -1.10 |
| RGD1566265 | *similar to RIKEN cDNA 2610002M06* | ENSRNOT00000003188 | 0.0108 | 35.28 | -1.10 |
| Ccdc55 | *coiled-coil domain containing 55* | NM_001037189 | 0.0311 | 47.72 | -1.10 |
| Olr12 | *olfactory receptor 12* | NM_001000540 | 0.0169 | 40.47 | -1.10 |
| Fth1 | *ferritin, heavy polypeptide 1* | BC081845 | 0.0499 | 54.56 | -1.10 |
| RGD1565496 | *similar to Butyrate-induced transcript 1* | NM_001106831 | 0.0012 | 17.72 | -1.10 |
| Cby1 | *chibby homolog 1 (Drosophila)* | NM_145676 | 0.0402 | 50.52 | -1.10 |
| Mxi1 | *MAX interactor 1* | NM_013160 | 0.0182 | 40.47 | -1.10 |
| Bcl10 | *B-cell CLL* | NM_031328 | 0.0158 | 40.47 | -1.10 |
| Gucy1b3 | *guanylate cyclase 1, soluble, beta 3* | NM_012769 | 0.0039 | 25.15 | -1.10 |
| Ankrd40 | *ankyrin repeat domain 40* | NM_001134699 | 0.0006 | 12.18 | -1.10 |
| Ccdc85a | *coiled-coil domain containing 85A* | NM_001191553 | 0.0410 | 50.52 | -1.10 |
| Fem1a | *fem-1 homolog a (C. elegans)* | NM_001025706 | 0.0033 | 25.15 | -1.10 |
| Tm4sf20 | *transmembrane 4 L six family member 20* | NM_001109618 | 0.0203 | 43.10 | -1.10 |
| Limd1 | *LIM domains containing 1* | NM_001112737 | 0.0159 | 40.47 | -1.09 |
| Gal | *galanin prepropeptide* | NM_033237 | 0.0476 | 54.56 | -1.09 |
| Snx19 | *sorting nexin 19* | NM_001108131 | 0.0076 | 32.49 | -1.09 |
| Trip4 | *thyroid hormone receptor interactor 4* | NM_001134981 | 0.0182 | 40.47 | -1.09 |
| Fam63b | *family with sequence similarity 63, member B* | ENSRNOT00000020089 | 0.0317 | 47.72 | -1.09 |
| Plekha1 | *Pleckstrin Homology Domain Containing, Family A (Phosphoinositide Binding Specific) Member 1* | NM_001079894 | 0.0101 | 35.28 | -1.09 |
| Zcwpw1 | *zinc finger, CW-type with PWWP domain 1* | ENSRNOT00000032838 | 0.0355 | 50.52 | -1.09 |
| Alg11 | *asparagine-linked glycosylation 11, alpha-1,2-mannosyltransferase* | NM_001108401 | 0.0015 | 20.07 | -1.09 |
| Olr473 | *olfactory receptor 473* | NM_001000302 | 0.0460 | 54.56 | -1.09 |
| Rbm34 | *RNA binding motif protein 34* | NM_001014015 | 0.0493 | 54.56 | -1.09 |
| RGD1561843 | *ribosomal protein L23a pseudogene* | ENSRNOT00000045791 | 0.0263 | 47.72 | -1.09 |
| LOC100362226 | *ribosomal protein L23-like* | ENSRNOT00000032589 | 0.0198 | 43.10 | -1.09 |
| Mrpl15 | *mitochondrial ribosomal protein L15* | NM_001106633 | 0.0189 | 40.47 | -1.09 |
| Dusp4 | *dual specificity phosphatase 4* | NM_022199 | 0.0083 | 32.49 | -1.09 |
| Atg4c | *ATG4 autophagy related 4 homolog C (S. cerevisiae)* | NM_001107948 | 0.0059 | 27.96 | -1.09 |
| RGD1310827 | *similar to RIKEN cDNA 1200009O22; EST AI316813* | NM_001034010 | 0.0222 | 43.10 | -1.09 |
| Nat8l | *N-acetyltransferase 8-like* | NM_001191681 | 0.0226 | 43.10 | -1.09 |
| B4galt4 | *UDP-Gal:BetaGlcNAc Beta 1,4- Galactosyltransferase, Polypeptide 4* | NM_001012018 | 0.0134 | 37.73 | -1.09 |
| Crnkl1 | *crooked neck pre-mRNA splicing factor-like 1 (Drosophila)* | NM_053797 | 0.0095 | 32.49 | -1.09 |
| Insig2 | *insulin induced gene 2* | NM_178091 | 0.0037 | 25.15 | -1.09 |
| LOC691543 | *hypothetical protein LOC691543* | NM_001109645 | 0.0284 | 47.72 | -1.09 |
| Pkia | *protein kinase (cAMP-dependent, catalytic) inhibitor alpha* | NM_053772 | 0.0100 | 35.28 | -1.09 |
| Sema5a | *Sema Domain, Seven Thrombospondin Repeats (Type 1 And Type 1-Like), Transmembrane Domain (TM) And Short Cytoplasmic Domain, (Semaphorin) 5A* | NM_001107659 | 0.0080 | 32.49 | -1.09 |
| Cbx4 | *chromobox homolog 4 (Pc class homolog, Drosophila)* | XM_576815 | 0.0300 | 47.72 | -1.09 |
| RGD1306613 | *similar to RIKEN cDNA 1600012F09* | NM_001107356 | 0.0475 | 54.56 | -1.09 |
| Ube2cbp | *ubiquitin-conjugating enzyme E2C binding protein* | NM_001039610 | 0.0257 | 43.10 | -1.09 |
| Bmi1 | *Bmi1 polycomb ring finger oncogene* | NM_001107368 | 0.0049 | 25.15 | -1.09 |
| Erbb3 | *v-erb-b2 erythroblastic leukemia viral oncogene homolog 3* | NM_017218 | 0.0405 | 50.52 | -1.09 |
| Cebpb | *CCAAT* | NM_024125 | 0.0226 | 43.10 | -1.09 |
| Ndufa6 | *NADH dehydrogenase (ubiquinone) 1 alpha subcomplex, 6* | NM_001130505 | 0.0268 | 47.72 | -1.09 |
| RGD1563945 | *similar to mKIAA0215 protein* | ENSRNOT00000061140 | 0.0034 | 25.15 | -1.09 |
| RGD1304694 | *similar to CG9646-PA* | ENSRNOT00000042070 | 0.0065 | 27.96 | -1.09 |
| Pck1 | *phosphoenolpyruvate carboxykinase 1 (soluble)* | NM_198780 | 0.0209 | 43.10 | -1.09 |
| Mtrf1l | *mitochondrial translational release factor 1-like* | NM_001025723 | 0.0140 | 37.73 | -1.09 |
| Pim3 | *pim-3 oncogene* | NM_022602 | 0.0061 | 27.96 | -1.09 |
| Fam163b | *family with sequence similarity 163, member B* | NM_001109458 | 0.0328 | 47.72 | -1.09 |
| Cript | *cysteine-rich PDZ-binding protein* | NM_019907 | 0.0074 | 29.67 | -1.09 |
| Ccdc51 | *coiled-coil domain containing 51* | NM_001014098 | 0.0381 | 50.52 | -1.09 |
| Tex11 | *testis expressed 11* | ENSRNOT00000041731 | 0.0164 | 40.47 | -1.09 |
| Ptgr2 | *prostaglandin reductase 2* | NM_001015009 | 0.0163 | 40.47 | -1.09 |
| Kbtbd11 | *kelch repeat and BTB (POZ) domain containing 11* | NM_001107326 | 0.0275 | 47.72 | -1.09 |
| Cdh13 | *cadherin 13* | NM_138889 | 0.0385 | 50.52 | -1.09 |
| LOC691024 | *similar to Protein C9orf25 homolog* | NM_001109616 | 0.0017 | 20.52 | -1.09 |
| Spon1 | *spondin 1, extracellular matrix protein* | NM_172067 | 0.0288 | 47.72 | -1.09 |
| LOC499900 | *similar to Zinc finger protein 133* | BC079070 | 0.0190 | 40.47 | -1.09 |
| Mosc2 | *MOCO sulphurase C-terminal domain containing 2* | NM_134410 | 0.0271 | 47.72 | -1.09 |
| Tmem168 | *transmembrane protein 168* | NM_001014054 | 0.0141 | 37.73 | -1.09 |
| LOC498735 | *similar to hypothetical protein MGC43581* | ENSRNOT00000022369 | 0.0454 | 54.56 | -1.09 |
| Cnot8 | *CCR4-NOT transcription complex, subunit 8* | NM_001008382 | 0.0168 | 40.47 | -1.09 |
| Ccdc109a | *coiled-coil domain containing 109A* | NM_001106398 | 0.0327 | 47.72 | -1.09 |
| Ckmt1 | *creatine kinase, mitochondrial 1* | NM_001012738 | 0.0156 | 37.73 | -1.09 |
| Blmh | *bleomycin hydrolase* | NM_001034163 | 0.0465 | 54.56 | -1.09 |
| Thumpd3 | *THUMP domain containing 3* | NM_001170546 | 0.0500 | 54.56 | -1.09 |
| Sfxn5 | *sideroflexin 5* | NM_153298 | 0.0029 | 25.15 | -1.09 |
| Zwilch | *Zwilch, kinetochore associated, homolog (Drosophila)* | ENSRNOT00000012404 | 0.0423 | 50.52 | -1.09 |
| Ndufa5 | *NADH dehydrogenase (ubiquinone) 1 alpha subcomplex 5* | NM_012985 | 0.0030 | 25.15 | -1.08 |
| Reg3a | *regenerating islet-derived 3 alpha* | NM_172077 | 0.0237 | 43.10 | -1.08 |
| Adamts18 | *ADAM metallopeptidase with thrombospondin type 1 motif* | NM_001191944 | 0.0427 | 50.52 | -1.08 |
| Fam84a | *family with sequence similarity 84, member A* | NM_001127299 | 0.0123 | 35.28 | -1.08 |
| Zfp189 | *zinc finger protein 189* | NM_001107930 | 0.0338 | 47.72 | -1.08 |
| Prr18 | *proline rich region 18* | NM_001108464 | 0.0369 | 50.52 | -1.08 |
| Spdye4 | *speedy homolog E4 (Xenopus laevis)* | NM_001034153 | 0.0366 | 50.52 | -1.08 |
| Ap1ar | *adaptor-related protein complex 1 associated regulatory protein* | NM_001191850 | 0.0235 | 43.10 | -1.08 |
| Glt1d1 | *glycosyltransferase 1 domain containing 1* | ENSRNOT00000064526 | 0.0460 | 54.56 | -1.08 |
| Rps15a | *ribosomal protein S15a* | NM_053982 | 0.0095 | 32.49 | -1.08 |
| Dck | *deoxycytidine kinase* | NM_024158 | 0.0318 | 47.72 | -1.08 |
| Cacng6 | *calcium channel, voltage-dependent, gamma subunit 6* | NM_080694 | 0.0423 | 50.52 | -1.08 |
| Hnrnpa3 | *heterogeneous nuclear ribonucleoprotein A3* | NM_001111294 | 0.0363 | 50.52 | -1.08 |
| Snrnp25 | *small Nuclear Ribonucleoprotein 25kDa (U11/U12)* | ENSRNOT00000067079 | 0.0189 | 40.47 | -1.08 |
| Snx30 | *sorting nexin family member 30* | NM_001106651 | 0.0051 | 25.15 | -1.08 |
| B4galt2 | *UDP-Gal:BetaGlcNAc Beta 1,4- Galactosyltransferase, Polypeptide 2* | NM_001107965 | 0.0197 | 43.10 | -1.08 |
| Gabra4 | *gamma-aminobutyric acid (GABA) A receptor, alpha 4* | NM_080587 | 0.0085 | 32.49 | -1.08 |
| Pdk1 | *pyruvate dehydrogenase kinase, isozyme 1* | NM_053826 | 0.0248 | 43.10 | -1.08 |
| Pigz | *phosphatidylinositol glycan anchor biosynthesis, class Z* | NM_001109525 | 0.0158 | 40.47 | -1.08 |
| Mxi1 | *MAX interactor 1* | NM_013160 | 0.0186 | 40.47 | -1.08 |
| Prpf18 | *PRP18 pre-mRNA processing factor 18 homolog (S. cerevisiae)* | NM_138523 | 0.0032 | 25.15 | -1.08 |
| Map2k1ip1 | *mitogen-activated protein kinase kinase 1 interacting protein 1* | NM_001008375 | 0.0450 | 54.56 | -1.08 |
| Pik3cb | *phosphoinositide-3-kinase, catalytic, beta polypeptide* | NM_053481 | 0.0058 | 27.96 | -1.08 |
| Ssr3 | *signal sequence receptor, gamma* | NM_031120 | 0.0154 | 37.73 | -1.08 |
| Fam19a2 | *family with sequence similarity 19 (chemokine)* | ENSRNOT00000005537 | 0.0382 | 50.52 | -1.08 |
| Cgref1 | *cell growth regulator with EF hand domain 1* | NM_139087 | 0.0195 | 43.10 | -1.08 |
| Hdac2 | *histone deacetylase 2* | NM_053447 | 0.0044 | 25.15 | -1.08 |
| Cdh6 | *cadherin 6* | NM_012927 | 0.0398 | 50.52 | -1.08 |
| Stradb | *STE20-related kinase adaptor beta* | NM_001109307 | 0.0155 | 37.73 | -1.08 |
| Fam181b | *family with sequence similarity 181, member B* | BC158703 | 0.0059 | 27.96 | -1.08 |
| Nf2 | *neurofibromin 2 (merlin)* | NM_013193 | 0.0410 | 50.52 | -1.08 |
| Chic2 | *cysteine-rich hydrophobic domain 2* | NM_001105736 | 0.0028 | 23.21 | -1.08 |
| RGD1566215 | *similar to Coatomer gamma-2 subunit* | NM_001106929 | 0.0409 | 50.52 | -1.08 |
| Kcns2 | *potassium voltage-gated channel, delayed-rectifier, subfamily S, member 2* | NM_023966 | 0.0438 | 50.52 | -1.08 |
| Tbx6 | *T-box 6* | NM_001108920 | 0.0487 | 54.56 | -1.08 |
| LOC100361579 | *prolactin family 5, subfamily a, member 2* | ENSRNOT00000059756 | 0.0348 | 50.52 | -1.08 |
| RGD1310951 | *similar to RIKEN cDNA E130308A19* | ENSRNOT00000022866 | 0.0069 | 29.67 | -1.08 |
| Slc25a25 | *Solute Carrier Family 25 (Mitochondrial Carrier; Phosphate Carrier), Member 25* | NM_145677 | 0.0175 | 40.47 | -1.08 |
| Dock11 | *dedicator of cytokinesis 11* | NM_001191760 | 0.0324 | 47.72 | -1.08 |
| Olr1472 | *olfactory receptor 1472* | NM_001000944 | 0.0427 | 50.52 | -1.08 |
| Kcna3 | *potassium voltage-gated channel, shaker-related subfamily* | NM_019270 | 0.0142 | 37.73 | -1.08 |
| Gpr150 | *G protein-coupled receptor 150* | NM_001109173 | 0.0412 | 50.52 | -1.08 |
| Galntl6 | *Polypeptide N-Acetylgalactosaminyltransferase-Like 6* | NM_001135756 | 0.0148 | 37.73 | -1.08 |
| Bhlhe41 | *basic helix-loop-helix family, member e41* | AF009329 | 0.0234 | 43.10 | -1.08 |
| Gabrb2 | *gamma-aminobutyric acid (GABA) A receptor, beta 2* | NM_012957 | 0.0065 | 27.96 | -1.08 |
| Zpbp | *zona pellucida binding protein* | NM_001025139 | 0.0355 | 50.52 | -1.08 |
| Sptlc2 | *serine palmitoyltransferase, long chain base subunit 2* | NM_001037097 | 0.0093 | 32.49 | -1.08 |
| Riok1 | *RIO kinase 1 (yeast)* | NM_001100511 | 0.0125 | 35.28 | -1.08 |
| LOC690358 | *similar to PR domain containing 11* | ENSRNOT00000011542 | 0.0108 | 35.28 | -1.08 |
| Fastkd3 | *FAST kinase domains 3* | NM_001082574 | 0.0266 | 47.72 | -1.08 |
| RGD1565410 | *similar to Ly6-C antigen gene* | BC158674 | 0.0464 | 54.56 | -1.08 |
| RragB | *Ras-related GTP binding B* | NM_053972 | 0.0415 | 50.52 | -1.08 |
| Slc27a4 | *solute carrier family 27 (fatty acid transporter), member 4* | NM_001100706 | 0.0115 | 35.28 | -1.08 |
| Ptprv | *protein tyrosine phosphatase, receptor type, V* | NM_033099 | 0.0206 | 43.10 | -1.08 |
| Aatk | *apoptosis-associated tyrosine kinase* | NM_001168703 | 0.0013 | 17.72 | -1.08 |
| Fam49b | *family with sequence similarity 49, member B* | BC166469 | 0.0027 | 23.21 | -1.08 |
| St18 | *suppression of tumorigenicity 18* | NM_153310 | 0.0427 | 50.52 | -1.08 |
| Gad1 | *glutamate decarboxylase 1* | NM_017007 | 0.0284 | 47.72 | -1.08 |
| Cadm4 | *cell adhesion molecule 4* | NM_001047107 | 0.0414 | 50.52 | -1.08 |
| Taf9b | *TAF9B RNA Polymerase II, TATA Box Binding Protein (TBP)-Associated Factor, 31kDa* | NM_133615 | 0.0353 | 50.52 | -1.08 |
| Rragd | *Ras-related GTP binding D* | NM_001106641 | 0.0042 | 25.15 | -1.08 |
| Steap3 | *STEAP family member 3* | NM_133314 | 0.0346 | 50.52 | -1.08 |
| Herc4 | *hect domain and RLD 4* | NM_001012074 | 0.0164 | 40.47 | -1.08 |
| Cirh1a | *cirrhosis, autosomal recessive 1A* | NM_001009640 | 0.0132 | 37.73 | -1.08 |
| Pak1 | *p21 Protein (Cdc42/Rac)-Activated Kinase 1* | NM_017198 | 0.0321 | 47.72 | -1.08 |
| RGD1359460 | *MMR_HSR1 domain containing protein RGD1359460* | NM_001006959 | 0.0492 | 54.56 | -1.08 |
| Naprt1 | *nicotinate phosphoribosyltransferase domain containing 1* | NM_207609 | 0.0111 | 35.28 | -1.08 |
| Snrnp27 | *small nuclear ribonucleoprotein 27kDa (U4)* | NM_001108636 | 0.0375 | 50.52 | -1.08 |
| Smc4 | *structural maintenance of chromosomes 4* | NM_001037185 | 0.0457 | 54.56 | -1.08 |
| Zfp161 | *zinc finger protein 161* | NM_172325 | 0.0116 | 35.28 | -1.08 |
| Gpt2 | *Glutamic Pyruvate Transaminase (Alanine Aminotransferase) 2* | NM_001012057 | 0.0225 | 43.10 | -1.08 |
| Smcr7l | *Smith-Magenis syndrome chromosome region, candidate 7-like* | NM_001007709 | 0.0321 | 47.72 | -1.08 |
| Btbd3 | *BTB (POZ) domain containing 3* | NM_001107782 | 0.0303 | 47.72 | -1.08 |
| Slc35f1 | *solute carrier family 35, member F1* | NM_001109338 | 0.0491 | 54.56 | -1.08 |
| RGD1310597 | *similar to RIKEN cDNA 1200014M14* | NM_001025647 | 0.0041 | 25.15 | -1.08 |
| Utp6 | *UTP6, Small Subunit (SSU) Processome Component, Homolog (Yeast)* | ENSRNOT00000019044 | 0.0173 | 40.47 | -1.08 |
| Mpped2 | *metallophosphoesterase domain containing 2* | NM_198778 | 0.0430 | 50.52 | -1.08 |
| KIFC2 | *kinesin family member C2* | NM_198752 | 0.0038 | 25.15 | -1.08 |
| Wee1 | *wee 1 homolog (S. pombe)* | NM_001012742 | 0.0228 | 43.10 | -1.08 |
| Arhgap27 | *Rho GTPase activating protein 27* | NM_198759 | 0.0371 | 50.52 | -1.08 |
| Ensa | *endosulfine alpha* | NM_001033974 | 0.0241 | 43.10 | -1.08 |
| Tjp2 | *tight junction protein 2* | NM_053773 | 0.0237 | 43.10 | -1.08 |
| Mxi1 | *MAX interactor 1* | NM_013160 | 0.0292 | 47.72 | -1.08 |
| RGD1564615 | *similar to mKIAA0241 protein* | ENSRNOT00000018425 | 0.0088 | 32.49 | -1.08 |
| Nadsyn1 | *NAD synthetase 1* | NM_181480 | 0.0396 | 50.52 | -1.08 |
| Hnrnpa3 | *heterogeneous nuclear ribonucleoprotein A3* | NM_001111295 | 0.0396 | 50.52 | -1.08 |
| Asb1 | *ankyrin repeat and SOCS box-containing 1* | NM_001108232 | 0.0131 | 37.73 | -1.08 |
| Atp6v1a | *ATPase, H+ transporting, lysosomal V1 subunit A* | NM_001108318 | 0.0127 | 35.28 | -1.08 |
| Prkar2a | *protein kinase, cAMP dependent regulatory, type II alpha* | NM_019264 | 0.0209 | 43.10 | -1.07 |
| Tmem49 | *transmembrane protein 49* | NM_138839 | 0.0136 | 37.73 | -1.07 |
| Sec14l1 | *SEC14-like 1 (S. cerevisiae)* | NM_001108309 | 0.0403 | 50.52 | -1.07 |
| Acbd4 | *acyl-Coenzyme A binding domain containing 4* | NM_001012013 | 0.0203 | 43.10 | -1.07 |
| Myl1 | *myosin, light polypeptide 1* | NM_001077656 | 0.0484 | 54.56 | -1.07 |
| Fam104a | *family with sequence similarity 104, member A* | NM_001034958 | 0.0304 | 47.72 | -1.07 |
| Aplp1 | *amyloid beta (A4) precursor-like protein 1* | NM_001100802 | 0.0002 | 0.00 | -1.07 |
| Srrp | *serine-arginine repressor protein* | NM_001135711 | 0.0428 | 50.52 | -1.07 |
| Egln1 | *EGL nine homolog 1 (C. elegans)* | AY228140 | 0.0259 | 47.72 | -1.07 |
| Kif26b | *kinesin family member 26B* | NM_001109079 | 0.0481 | 54.56 | -1.07 |
| Sephs2 | *selenophosphate synthetase 2* | NM_001079889 | 0.0154 | 37.73 | -1.07 |
| Suox | *sulfite oxidase* | NM_031127 | 0.0201 | 43.10 | -1.07 |
| Cabp7 | *calcium binding protein 7* | NM_001007730 | 0.0340 | 47.72 | -1.07 |
| Fgr | *Gardner-Rasheed feline sarcoma viral (v-fgr) oncogene homolog* | NM_024145 | 0.0141 | 37.73 | -1.07 |
| Pc | *pyruvate carboxylase* | NM_012744 | 0.0100 | 35.28 | -1.07 |
| Nudt15 | *nudix (nucleoside diphosphate linked moiety X)-type motif 15* | NM_001106049 | 0.0385 | 50.52 | -1.07 |
| Cbx7 | *chromobox homolog 7* | NM_199117 | 0.0353 | 50.52 | -1.07 |
| Gnl3l | *guanine nucleotide binding protein-like 3 (nucleolar)-like* | NM_001081958 | 0.0484 | 54.56 | -1.07 |
| Galntl6 | *Polypeptide N-Acetylgalactosaminyltransferase-Like 6* | NM_001135756 | 0.0276 | 47.72 | -1.07 |
| Fgd3 | *FYVE, RhoGEF and PH domain containing 3* | NM_001108409 | 0.0489 | 54.56 | -1.07 |
| Uprt | *Uracil Phosphoribosyltransferase (FUR1) Homolog (S. Cerevisiae)* | ENSRNOT00000033494 | 0.0308 | 47.72 | -1.07 |
| Hapln1 | *hyaluronan and proteoglycan link protein 1* | NM_019189 | 0.0482 | 54.56 | -1.07 |
| Fam134b | *family with sequence similarity 134, member B* | NM_001034912 | 0.0445 | 50.52 | -1.07 |
| Fbxo28 | *F-box protein 28* | NM_001107203 | 0.0039 | 25.15 | -1.07 |
| Ntn1 | *netrin 1* | NM_053731 | 0.0240 | 43.10 | -1.07 |
| Slc2a13 | *Solute Carrier Family 2 (Facilitated Glucose Transporter), Member 13* | NM_133611 | 0.0024 | 23.21 | -1.07 |
| Mboat2 | *membrane bound O-acyltransferase domain containing 2* | NM_001108016 | 0.0239 | 43.10 | -1.07 |
| Ero1l | *ERO1-like (S. cerevisiae)* | NM_138528 | 0.0100 | 35.28 | -1.07 |
| Hecw2 | *HECT, C2 and WW domain containing E3 ubiquitin protein* | NM_001108218 | 0.0036 | 25.15 | -1.07 |
| Mta2 | *metastasis associated 1 family, member 2* | NM_001100740 | 0.0129 | 37.73 | -1.07 |
| Pctk2 | *PCTAIRE protein kinase 2* | NM_001108082 | 0.0182 | 40.47 | -1.07 |
| Olig2 | *oligodendrocyte lineage transcription factor 2* | NM_001100557 | 0.0244 | 43.10 | -1.07 |
| Zfp91 | *zinc finger protein 91* | NM_001169120 | 0.0033 | 25.15 | -1.07 |
| Pcdhb18 | *protocadherin beta 18* | XM_001065313 | 0.0289 | 47.72 | -1.07 |
| RGD1564943 | *similar to 4930429A08Rik protein* | NM_001134628 | 0.0416 | 50.52 | -1.07 |
| Add1 | *adducin 1 (alpha)* | NM_016990 | 0.0004 | 9.75 | -1.07 |
| Cspg5 | *chondroitin sulfate proteoglycan 5* | NM_133652 | 0.0019 | 23.21 | -1.07 |
| Lrtm2 | *leucine-rich repeats and transmembrane domains 2* | NM_001109430 | 0.0189 | 40.47 | -1.07 |
| Mtmr2 | *myotubularin related protein 2* | NM_001108123 | 0.0338 | 47.72 | -1.07 |
| Pcdhb19 | *protocadherin beta 19* | ENSRNOT00000060484 | 0.0447 | 50.52 | -1.07 |
| Psat1 | *phosphoserine aminotransferase 1* | NM_198738 | 0.0277 | 47.72 | -1.07 |
| Has1 | *hyaluronan synthase 1* | NM_172323 | 0.0453 | 54.56 | -1.07 |
| Crebbp | *CREB binding protein* | NM_133381 | 0.0178 | 40.47 | -1.07 |
| Git1 | *G protein-coupled receptor kinase interacting ArfGAP 1* | NM_031814 | 0.0119 | 35.28 | -1.07 |
| Rngtt | *RNA guanylyltransferase and 5'-phosphatase* | NM_001107923 | 0.0177 | 40.47 | -1.07 |
| Exoc8 | *exocyst complex component 8* | NM_139043 | 0.0181 | 40.47 | -1.07 |
| Rad51ap1 | *RAD51 associated protein 1* | NM_001079711 | 0.0405 | 50.52 | -1.07 |
| Gzf1 | *GDNF-inducible zinc finger protein 1* | NM_001107788 | 0.0491 | 54.56 | -1.07 |
| Mtpap | *mitochondrial poly(A) polymerase* | NM_001107359 | 0.0250 | 43.10 | -1.07 |
| Lclat1 | *lysocardiolipin acyltransferase 1* | ENSRNOT00000043641 | 0.0035 | 25.15 | -1.07 |
| Olr23 | *olfactory receptor 23* | NM_001000119 | 0.0447 | 50.52 | -1.07 |
| Samd12 | *sterile alpha motif domain containing 12* | NM_001130562 | 0.0090 | 32.49 | -1.07 |
| Cdc2l6 | *cell division cycle 2-like 6 (CDK8-like)* | NM_001107634 | 0.0147 | 37.73 | -1.07 |
| Gpr137b | *G protein-coupled receptor 137B* | NM_001105978 | 0.0108 | 35.28 | -1.07 |
| Coil | *coilin* | NM_017360 | 0.0177 | 40.47 | -1.07 |
| Kars | *lysyl-tRNA synthetase* | NM_001006967 | 0.0208 | 43.10 | -1.07 |
| Dnajb14 | *DnaJ (Hsp40) homolog, subfamily B, member 14* | NM_001109193 | 0.0011 | 17.72 | -1.07 |
| Sult5a1 | *sulfotransferase family 5A, member 1* | NM_001106194 | 0.0345 | 47.72 | -1.07 |
| Asb6 | *ankyrin repeat and SOCS box-containing 6* | NM_001011963 | 0.0105 | 35.28 | -1.07 |
| Rpl26 | *ribosomal protein L26* | NM_001105788 | 0.0493 | 54.56 | -1.07 |
| Fancf | *Fanconi anemia, complementation group F* | ENSRNOT00000032922 | 0.0374 | 50.52 | -1.07 |
| Dcps | *decapping enzyme, scavenger* | NM_153302 | 0.0484 | 54.56 | -1.07 |
| Tmem86a | *transmembrane protein 86A* | NM_001135016 | 0.0380 | 50.52 | -1.07 |
| LOC100361606 | *rCG39624-like* | ENSRNOT00000024639 | 0.0381 | 50.52 | -1.07 |
| Sertad2 | *SERTA domain containing 2* | NM_001024903 | 0.0285 | 47.72 | -1.07 |
| Rragc | *Ras-related GTP binding C* | NM_001048184 | 0.0438 | 50.52 | -1.07 |
| Mfsd1 | *major facilitator superfamily domain containing 1* | NM_001191847 | 0.0177 | 40.47 | -1.07 |
| LOC100158225 | *hypothetical protein LOC100158225* | NM_001127606 | 0.0349 | 50.52 | -1.07 |
| Impad1 | *inositol monophosphatase domain containing 1* | NM_001008772 | 0.0122 | 35.28 | -1.07 |
| Smek2 | *SMEK homolog 2, suppressor of mek1 (Dictyostelium)* | NM_001108367 | 0.0054 | 27.96 | -1.07 |
| Smyd3 | *SET and MYND domain containing 3* | NM_001025762 | 0.0297 | 47.72 | -1.07 |
| Magmas | *mitochondria-associated protein involved in granulocytosis* | NM_001100136 | 0.0200 | 43.10 | -1.07 |
| RGD1307704 | *similar to RIKEN cDNA 2410016O06* | NM_001108040 | 0.0402 | 50.52 | -1.07 |
| St3gal4 | *ST3 beta-galactoside alpha-2,3-sialyltransferase 4* | NM_203337 | 0.0471 | 54.56 | -1.07 |
| Cntn2 | *contactin 2 (axonal)* | NM_012884 | 0.0471 | 54.56 | -1.07 |
| Mgll | *monoglyceride lipase* | NM_138502 | 0.0335 | 47.72 | -1.07 |
| Lrrc59 | *leucine rich repeat containing 59* | NM_001008280 | 0.0294 | 47.72 | -1.07 |
| Traip | *TRAF-interacting protein* | NM_001109004 | 0.0186 | 40.47 | -1.07 |
| Plekhg1 | *Pleckstrin Homology Domain Containing, Family G (With RhoGef Domain) Member 1* | NM_001190999 | 0.0497 | 54.56 | -1.07 |
| Hnrnpa3 | *heterogeneous nuclear ribonucleoprotein A3* | NM_001111294 | 0.0479 | 54.56 | -1.07 |
| Supt16h | *suppressor of Ty 16 homolog (S. cerevisiae)* | NM_001107261 | 0.0042 | 25.15 | -1.07 |
| Letmd1 | *LETM1 domain containing 1* | NM_001122781 | 0.0055 | 27.96 | -1.07 |
| Slc1a3 | *Solute Carrier Family 1 (Glial High Affinity Glutamate Transporter), Member 3* | NM_019225 | 0.0103 | 35.28 | -1.07 |
| Tardbp | *TAR DNA binding protein* | NM_001011979 | 0.0334 | 47.72 | -1.07 |
| Sept2 | *septin 2* | NM_057148 | 0.0130 | 37.73 | -1.07 |
| Lancl1 | *LanC lantibiotic synthetase component C-like 1 (bacterial)* | NM_053723 | 0.0101 | 35.28 | -1.07 |
| Gpr84 | *G protein-coupled receptor 84* | NM_001109509 | 0.0304 | 47.72 | -1.07 |
| Lkap | *limkain b1* | NM_133421 | 0.0136 | 37.73 | -1.07 |
| Fancg | *Fanconi anemia, complementation group G* | ENSRNOT00000013903 | 0.0296 | 47.72 | -1.07 |
| Ttyh3 | *tweety homolog 3 (Drosophila)* | NM_001107124 | 0.0087 | 32.49 | -1.07 |
| Vps4a | *vacuolar protein sorting 4 homolog A (S. cerevisiae)* | NM_145678 | 0.0084 | 32.49 | -1.07 |
| RGD1560691 | *similar to calcium* | NM_001107365 | 0.0171 | 40.47 | -1.07 |
| Mfhas1 | *malignant fibrous histiocytoma amplified sequence 1* | NM_001107316 | 0.0268 | 47.72 | -1.07 |
| Cyp7b1 | *cytochrome P450, family 7, subfamily b, polypeptide 1* | NM_019138 | 0.0247 | 43.10 | -1.07 |
| Fdxacb1 | *ferredoxin-fold anticodon binding domain containing 1* | NM_001108145 | 0.0388 | 50.52 | -1.07 |
| Hiatl1 | *hippocampus abundant transcript-like 1* | NM_001107334 | 0.0228 | 43.10 | -1.07 |
| RGD1306119 | *similar to transcriptional regulating protein 132* | NM_001169116 | 0.0415 | 50.52 | -1.07 |
| Med28 | *mediator complex subunit 28* | NM_001107217 | 0.0268 | 47.72 | -1.07 |
| Rmnd1 | *required for meiotic nuclear division 1 homolog (S. cerevisiae)* | NM_001040128 | 0.0150 | 37.73 | -1.07 |
| Itpkc | *inositol 1,4,5-trisphosphate 3-kinase C* | NM_178094 | 0.0363 | 50.52 | -1.07 |
| Creld1 | *cysteine-rich with EGF-like domains 1* | NM_001024783 | 0.0022 | 23.21 | -1.07 |
| Tspan5 | *tetraspanin 5* | NM_001004090 | 0.0343 | 47.72 | -1.07 |
| Elfn1 | *extracellular leucine-rich repeat and fibronectin type* | NM_001105913 | 0.0114 | 35.28 | -1.07 |
| Rps23 | *ribosomal protein S23* | NM_078617 | 0.0412 | 50.52 | -1.07 |
| RGD1559896 | *similar to RIKEN cDNA 2310022B05* | NM_001109134 | 0.0099 | 35.28 | -1.07 |
| Morc3 | *MORC family CW-type zinc finger 3* | NM_001107109 | 0.0143 | 37.73 | -1.07 |
| Cwf19l1 | *CWF19-like 1, cell cycle control (S. pombe)* | NM_001108928 | 0.0110 | 35.28 | -1.07 |
| RGD1309437 | *similar to RIKEN cDNA 2610528E23* | BC089826 | 0.0369 | 50.52 | -1.07 |
| Atg5 | *ATG5 autophagy related 5 homolog (S. cerevisiae)* | NM_001014250 | 0.0214 | 43.10 | -1.07 |
| Golga7 | *golgi autoantigen, golgin subfamily a, 7* | NM_001007731 | 0.0119 | 35.28 | -1.07 |
| Pdgfc | *platelet derived growth factor C* | NM_031317 | 0.0416 | 50.52 | -1.07 |
| Ccdc72 | *coiled-coil domain containing 72* | NM_001126048 | 0.0278 | 47.72 | -1.07 |
| Esco1 | *establishment of cohesion 1 homolog 1 (S. cerevisiae)* | BC166441 | 0.0202 | 43.10 | -1.07 |
| Adcyap1r1 | *adenylate cyclase activating polypeptide 1 receptor 1* | NM_133511 | 0.0196 | 43.10 | -1.07 |
| Fktn | *fukutin* | NM_001108667 | 0.0381 | 50.52 | -1.06 |
| Tshz1 | *teashirt zinc finger homeobox 1* | ENSRNOT00000021750 | 0.0406 | 50.52 | -1.06 |
| Rps18 | *ribosomal protein S18* | NM_213557 | 0.0296 | 47.72 | -1.06 |
| RGD1560436 | *similar to hypothetical protein FLJ20508* | NM_001109264 | 0.0398 | 50.52 | -1.06 |
| Lzic | *leucine zipper and CTNNBIP1 domain containing* | NM_001013241 | 0.0470 | 54.56 | -1.06 |
| RGD1306746 | *similar to Hypothetical protein MGC25529* | NM_001107872 | 0.0074 | 29.67 | -1.06 |
| Mgam | *maltase-glucoamylase* | ENSRNOT00000052339 | 0.0148 | 37.73 | -1.06 |
| Snx25 | *sorting nexin 25* | ENSRNOT00000014828 | 0.0056 | 27.96 | -1.06 |
| Ddn | *dendrin* | NM_030993 | 0.0273 | 47.72 | -1.06 |
| RGD1310862 | *similar to adult retina protein* | ENSRNOT00000028186 | 0.0233 | 43.10 | -1.06 |
| Polr3d | *polymerase (RNA) III (DNA directed) polypeptide D* | NM_001031653 | 0.0295 | 47.72 | -1.06 |
| Mapk10 | *mitogen activated protein kinase 10* | NM_012806 | 0.0289 | 47.72 | -1.06 |
| Ppp2r3a | *Protein Phosphatase 2, Regulatory Subunit B'', Alpha* | NM_001012202 | 0.0482 | 54.56 | -1.06 |
| Sema6d | *Sema Domain, Transmembrane Domain (TM), And Cytoplasmic Domain, (Semaphorin) 6D* | NM_001107768 | 0.0462 | 54.56 | -1.06 |
| Fam134a | *family with sequence similarity 134, member A* | ENSRNOT00000057330 | 0.0159 | 40.47 | -1.06 |
| Pm20d2 | *peptidase M20 domain containing 2* | NM_001107922 | 0.0407 | 50.52 | -1.06 |
| Tfdp2 | *transcription factor Dp-2 (E2F dimerization partner 2)* | NM_001106847 | 0.0461 | 54.56 | -1.06 |
| Tmem103 | *transmembrane protein 103* | NM_001108782 | 0.0287 | 47.72 | -1.06 |
| Cacng4 | *calcium channel, voltage-dependent, gamma subunit 4* | NM_080692 | 0.0467 | 54.56 | -1.06 |
| Prmt6 | *protein arginine methyltransferase 6* | NM_001106466 | 0.0383 | 50.52 | -1.06 |
| Azin1 | *antizyme inhibitor 1* | NM_022585 | 0.0198 | 43.10 | -1.06 |
| LOC679974 | *similar to transcription elongation factor A* | ENSRNOT00000051516 | 0.0311 | 47.72 | -1.06 |
| Abat | *4-aminobutyrate aminotransferase* | NM_031003 | 0.0041 | 25.15 | -1.06 |
| Sox4 | *SRY (sex determining region Y)-box 4* | XM_344594 | 0.0052 | 27.96 | -1.06 |
| Lrp11 | *low density lipoprotein receptor-related protein 11* | NM_001106217 | 0.0024 | 23.21 | -1.06 |
| Taz | *tafazzin* | NM_001025748 | 0.0302 | 47.72 | -1.06 |
| Kif26b | *kinesin family member 26B* | NM_001109079 | 0.0242 | 43.10 | -1.06 |
| Sh3bp5 | *SH3-domain binding protein 5 (BTK-associated)* | NM_054011 | 0.0466 | 54.56 | -1.06 |
| LOC691468 | *similar to Zinc finger protein 84 (Zinc finge* | ENSRNOT00000001788 | 0.0108 | 35.28 | -1.06 |
| Nlrp5 | *NLR family, pyrin domain containing 5* | NM_001107474 | 0.0152 | 37.73 | -1.06 |
| Adcy2 | *adenylate cyclase 2 (brain)* | NM_031007 | 0.0036 | 25.15 | -1.06 |
| Hnrpll | *heterogeneous nuclear ribonucleoprotein L-like* | ENSRNOT00000009175 | 0.0055 | 27.96 | -1.06 |
| Adam23 | *ADAM metallopeptidase domain 23* | NM_001029899 | 0.0060 | 27.96 | -1.06 |
| Tmtc4 | *transmembrane and tetratricopeptide repeat containing 4* | NM_001134414 | 0.0065 | 27.96 | -1.06 |
| G3bp1 | *GTPase activating protein (SH3 domain) binding protein 1* | NM_133565 | 0.0499 | 54.56 | -1.06 |
| Nanos1 | *nanos homolog 1 (Drosophila)* | ENSRNOT00000036203 | 0.0076 | 32.49 | -1.06 |
| Ppp2r2d | *protein phosphatase 2, regulatory subunit B, delta isoform* | NM_144746 | 0.0367 | 50.52 | -1.06 |
| Glrb | *glycine receptor, beta* | NM_053296 | 0.0204 | 43.10 | -1.06 |
| Ranbp6 | *RAN binding protein 6* | NM_001107584 | 0.0297 | 47.72 | -1.06 |
| Ndrg3 | *N-myc downstream regulated gene 3* | NM_001013923 | 0.0329 | 47.72 | -1.06 |
| Ccdc8 | *coiled-coil domain containing 8* | NM_001009533 | 0.0297 | 47.72 | -1.06 |
| Cdc42se1 | *CDC42 small effector 1* | NM_001039044 | 0.0184 | 40.47 | -1.06 |
| Aldh5a1 | *aldehyde dehydrogenase 5 family, member A1* | ENSRNOT00000031384 | 0.0239 | 43.10 | -1.06 |
| Trpc4ap | *Transient Receptor Potential Cation Channel, Subfamily C, Member 4 Associated Protein* | NM_001100748 | 0.0085 | 32.49 | -1.06 |
| RGD1563349 | *similar to RIKEN cDNA 9330182L06* | NM_001109345 | 0.0340 | 47.72 | -1.06 |
| Cse1l | *CSE1 chromosome segregation 1-like (yeast)* | NM_001108607 | 0.0072 | 29.67 | -1.06 |
| Galntl6 | *Polypeptide N-Acetylgalactosaminyltransferase-Like 6* | NM_001135756 | 0.0210 | 43.10 | -1.06 |
| Psmd11 | *proteasome (prosome, macropain) 26S subunit, non-ATPase 11* | NM_001107027 | 0.0179 | 40.47 | -1.06 |
| Lrp12 | *low density lipoprotein-related protein 12* | NM_001134883 | 0.0500 | 54.56 | -1.06 |
| Nat13 | *N-acetyltransferase 13* | NM_001105881 | 0.0075 | 29.67 | -1.06 |
| RGD1305500 | *similar to hypothetical protein FLJ13188* | NM_001107448 | 0.0348 | 50.52 | -1.06 |
| Siglec5 | *sialic acid binding Ig-like lectin 5* | NM_001106249 | 0.0034 | 25.15 | -1.06 |
| Foxn3 | *forkhead box N3* | NM_001108047 | 0.0445 | 50.52 | -1.06 |
| Tsc22d2 | *TSC22 domain family, member 2* | NM_001191960 | 0.0279 | 47.72 | -1.06 |
| Cirbp | *cold inducible RNA binding protein* | NM_031147 | 0.0363 | 50.52 | -1.06 |
| Clptm1l | *CLPTM1-like* | NM_001108240 | 0.0116 | 35.28 | -1.06 |
| Ophn1 | *oligophrenin 1* | NM_001107848 | 0.0059 | 27.96 | -1.06 |
| Nampt | *nicotinamide phosphoribosyltransferase* | NM_177928 | 0.0061 | 27.96 | -1.06 |
| Mbp | *myelin basic protein* | NM_001025291 | 0.0203 | 43.10 | -1.06 |
| Brsk2 | *BR serine* | ENSRNOT00000027134 | 0.0194 | 43.10 | -1.06 |
| Pygo2 | *pygopus 2* | NM_001106447 | 0.0246 | 43.10 | -1.06 |
| Mtmr7 | *myotubularin related protein 7* | NM_001107312 | 0.0122 | 35.28 | -1.06 |
| Cx3cl1 | *chemokine (C-X3-C motif) ligand 1* | NM_134455 | 0.0171 | 40.47 | -1.06 |
| Sept11 | *septin 11* | NM_001107208 | 0.0314 | 47.72 | -1.06 |
| Itch | *itchy E3 ubiquitin protein ligase homolog (mouse)* | NM_001005887 | 0.0301 | 47.72 | -1.06 |
| RGD1303127 | *similar to hypothetical protein FLJ20436* | NM_001004244 | 0.0490 | 54.56 | -1.06 |
| Vps26b | *vacuolar protein sorting 26 homolog B (S. pombe)* | NM_001106809 | 0.0257 | 43.10 | -1.06 |
| B3galnt1 | *beta-1,3-N-acetylgalactosaminyltransferase 1* | NM_001013158 | 0.0497 | 54.56 | -1.06 |
| Fxr2 | *fragile X mental retardation, autosomal homolog 2* | NM_001100647 | 0.0018 | 20.52 | -1.06 |
| Ubl3 | *ubiquitin-like 3* | NM_001015030 | 0.0105 | 35.28 | -1.06 |
| Clcn4-2 | *chloride channel 4-2* | NM_022198 | 0.0167 | 40.47 | -1.06 |
| Rfx4 | *regulatory Factor X, 4 (Influences HLA Class II Expression)* | ENSRNOT00000059685 | 0.0441 | 50.52 | -1.06 |
| rnf141 | *ring finger protein 141* | NM_001001800 | 0.0244 | 43.10 | -1.06 |
| Elavl3 | *ELAV (embryonic lethal, abnormal vision, Drosophila)-like* | NM_172324 | 0.0213 | 43.10 | -1.06 |
| Nptxr | *neuronal pentraxin receptor* | NM_030841 | 0.0454 | 54.56 | -1.06 |
| Ssrp1 | *structure specific recognition protein 1* | NM_031121 | 0.0302 | 47.72 | -1.06 |
| Sf3b4 | *splicing factor 3b, subunit 4* | NM_001011951 | 0.0371 | 50.52 | -1.06 |
| Tacc1 | *transforming, acidic coiled-coil containing protein 1* | NM_001004107 | 0.0169 | 40.47 | -1.06 |
| Slc38a1 | *solute carrier family 38, member 1* | NM_138832 | 0.0151 | 37.73 | -1.06 |
| Rnf103 | *ring finger protein 103* | NM_053438 | 0.0264 | 47.72 | -1.06 |
| MGC72612 | *similar to expressed sequence AI449175* | NM_001009538 | 0.0402 | 50.52 | -1.06 |
| Camta1 | *similar to KIAA0833 protein* | NM_001195559 | 0.0233 | 43.10 | -1.06 |
| Cacng7 | *calcium channel, voltage-dependent, gamma subunit 7* | NM_080695 | 0.0224 | 43.10 | -1.06 |
| Mbtps1 | *membrane-bound transcription factor peptidase, site 1* | NM_053569 | 0.0065 | 27.96 | -1.06 |
| Mgat1 | *Mannosyl (Alpha-1,3-)-Glycoprotein Beta-1,2-N-Acetylglucosaminyltransferase* | NM_030861 | 0.0201 | 43.10 | -1.06 |
| Camkk1 | *Calcium/Calmodulin-Dependent Protein Kinase Kinase 1, Alpha* | NM_031662 | 0.0315 | 47.72 | -1.06 |
| Arl6ip1 | *ADP-ribosylation factor-like 6 interacting protein 1* | NM_198737 | 0.0084 | 32.49 | -1.06 |
| Nat15 | *N-acetyltransferase 15 (GCN5-related, putative)* | NM_001014226 | 0.0198 | 43.10 | -1.06 |
| Asb13 | *ankyrin repeat and SOCS box-containing 13* | NM_001108420 | 0.0162 | 40.47 | -1.06 |
| Caskin2 | *cask-interacting protein 2* | NM_001107065 | 0.0193 | 40.47 | -1.06 |
| Dgat1 | *diacylglycerol O-acyltransferase homolog 1 (mouse)* | NM_053437 | 0.0325 | 47.72 | -1.06 |
| Ttc9c | *tetratricopeptide repeat domain 9C* | NM_001007693 | 0.0236 | 43.10 | -1.06 |
| Tex2 | *testis expressed 2* | ENSRNOT00000055114 | 0.0184 | 40.47 | -1.06 |
| S100b | *S100 calcium binding protein B* | NM_013191 | 0.0422 | 50.52 | -1.06 |
| RGD1565784 | *RGD1565784* | NM_001109028 | 0.0333 | 47.72 | -1.06 |
| Scg3 | *secretogranin III* | NM_053856 | 0.0292 | 47.72 | -1.06 |
| Tbc1d14 | *TBC1 domain family, member 14* | NM_001113365 | 0.0111 | 35.28 | -1.06 |
| Rab23 | *RAB23, member RAS oncogene family* | NM_001109005 | 0.0408 | 50.52 | -1.06 |
| Scn1a | *sodium channel, voltage-gated, type I, alpha* | NM_030875 | 0.0188 | 40.47 | -1.06 |
| Cadps2 | *Ca++-dependent secretion activator 2* | ENSRNOT00000068404 | 0.0338 | 47.72 | -1.06 |
| Stmn1 | *stathmin 1* | NM_017166 | 0.0368 | 50.52 | -1.06 |
| Ndufa3 | *NADH dehydrogenase (ubiquinone) 1 alpha subcompliment 3* | ENSRNOT00000019141 | 0.0265 | 47.72 | -1.05 |
| Sh2d5 | *SH2 domain containing 5* | ENSRNOT00000067834 | 0.0038 | 25.15 | -1.05 |
| Cul2 | *cullin 2* | NM_001108417 | 0.0339 | 47.72 | -1.05 |
| Cdh22 | *cadherin 22* | NM_019161 | 0.0309 | 47.72 | -1.05 |
| Gpr37l1 | *G protein-coupled receptor 37-like 1* | NM_145784 | 0.0272 | 47.72 | -1.05 |
| Brd2 | *bromodomain containing 2* | NM_212495 | 0.0357 | 50.52 | -1.05 |
| Rhbdl1 | *rhomboid, veinlet-like 1 (Drosophila)* | NM_001191822 | 0.0178 | 40.47 | -1.05 |
| Actr2 | *ARP2 actin-related protein 2 homolog (yeast)* | NM_001009268 | 0.0120 | 35.28 | -1.05 |
| Adss | *adenylosuccinate synthase* | NM_001105975 | 0.0235 | 43.10 | -1.05 |
| Zmpste24 | *zinc metallopeptidase, STE24 homolog (S. cerevisiae)* | NM_001107974 | 0.0449 | 54.56 | -1.05 |
| Terf2 | *telomeric repeat binding factor 2* | NM_001108448 | 0.0130 | 37.73 | -1.05 |
| Lpcat4 | *lysophosphatidylcholine acyltransferase 4* | NM_001106494 | 0.0103 | 35.28 | -1.05 |
| Ptma | *prothymosin alpha* | NM_021740 | 0.0280 | 47.72 | -1.05 |
| Lrp3 | *low density lipoprotein receptor-related protein 3* | NM_053541 | 0.0279 | 47.72 | -1.05 |
| Mmd2 | *monocyte to macrophage differentiation-associated 2* | NM_001037217 | 0.0062 | 27.96 | -1.05 |
| Dennd5a | *DENN* | NM_001107546 | 0.0053 | 27.96 | -1.05 |
| RGD1565257 | *similar to zinc finger protein 650* | NM_001134550 | 0.0321 | 47.72 | -1.05 |
| Arhgap20 | *Rho GTPase activating protein 20* | NM_213629 | 0.0424 | 50.52 | -1.05 |
| Mon1a | *MON1 homolog A (yeast)* | NM_001126284 | 0.0084 | 32.49 | -1.05 |
| Atcay | *ataxia, cerebellar, Cayman type* | NM_001040190 | 0.0318 | 47.72 | -1.05 |
| RGD1559958 | *similar to RIKEN cDNA C030011O14 gene* | ENSRNOT00000016668 | 0.0044 | 25.15 | -1.05 |
| Tbc1d15 | *TBC1 domain family, member 15* | ENSRNOT00000005207 | 0.0098 | 35.28 | -1.05 |
| Gpr137 | *G protein-coupled receptor 137* | NM_001109562 | 0.0310 | 47.72 | -1.05 |
| Ppp1r2 | *protein phosphatase 1, regulatory (inhibitor) subunit 2* | NM_138823 | 0.0078 | 32.49 | -1.05 |
| RGD1560248 | *similar to formin-like 2 isoform B* | ENSRNOT00000006433 | 0.0212 | 43.10 | -1.05 |
| Pank3 | *pantothenate kinase 3* | NM_001108272 | 0.0104 | 35.28 | -1.05 |
| Mapkap1 | *mitogen-activated protein kinase associated protein 1* | NM_001011964 | 0.0123 | 35.28 | -1.05 |
| LOC685152 | *similar to Probable phospholipid-transporting* | ENSRNOT00000056606 | 0.0499 | 54.56 | -1.05 |
| Abcf1 | *ATP-binding cassette, sub-family F (GCN20), member 1* | NM_001109883 | 0.0251 | 43.10 | -1.05 |
| Ppap2b | *phosphatidic acid phosphatase type 2B* | NM_138905 | 0.0102 | 35.28 | -1.05 |
| Map7d2 | *MAP7 domain containing 2* | ENSRNOT00000045285 | 0.0420 | 50.52 | -1.05 |
| Rps6kb1 | *ribosomal protein S6 kinase, 70kDa, polypeptide 1* | NM_031985 | 0.0259 | 47.72 | -1.05 |
| Cpeb3 | *cytoplasmic polyadenylation element binding protein* | ENSRNOT00000047387 | 0.0116 | 35.28 | -1.05 |
| Rap1gds1 | *RAP1, GTP-GDP dissociation stimulator 1* | NM_001107728 | 0.0416 | 50.52 | -1.05 |
| Klhl26 | *kelch-like 26 (Drosophila)* | NM_001106075 | 0.0173 | 40.47 | -1.05 |
| Lrrc8a | *leucine rich repeat containing 8 family, member A* | NM_001024782 | 0.0227 | 43.10 | -1.05 |
| Nedd4l | *Neural Precursor Cell Expressed, Developmentally Down-Regulated 4-Like, E3 Ubiquitin Protein Ligase* | NM_001008300 | 0.0185 | 40.47 | -1.05 |
| Sept8 | *septin 8* | NM_001107002 | 0.0106 | 35.28 | -1.05 |
| Myst2 | *MYST histone acetyltransferase 2* | NM_181081 | 0.0190 | 40.47 | -1.05 |
| Slc25a23 | *Solute Carrier Family 25 (Mitochondrial Carrier; Phosphate Carrier), Member 23* | NM_001106873 | 0.0245 | 43.10 | -1.05 |
| Rnf215 | *ring finger protein 215* | NM_001107234 | 0.0379 | 50.52 | -1.05 |
| Sh3glb2 | *SH3-domain GRB2-like endophilin B2* | NM_001009692 | 0.0238 | 43.10 | -1.05 |
| Mrpl1 | *mitochondrial ribosomal protein L1* | NM_001105997 | 0.0320 | 47.72 | -1.05 |
| Dbndd1 | *Dysbindin (Dystrobrevin Binding Protein 1) Domain Containing 1* | NM_001014156 | 0.0329 | 47.72 | -1.05 |
| Rps10 | *ribosomal protein S10* | NM_031109 | 0.0330 | 47.72 | -1.05 |
| Gnb5 | *guanine nucleotide binding protein (G protein), beta 5* | NM_031770 | 0.0330 | 47.72 | -1.05 |
| Cbx5 | *chromobox homolog 5 (HP1 alpha homolog, Drosophila)* | NM_001106797 | 0.0433 | 50.52 | -1.05 |
| Pitpnc1 | *Phosphatidylinositol Transfer Protein, Cytoplasmic 1* | ENSRNOT00000021498 | 0.0271 | 47.72 | -1.05 |
| Rps6ka5 | *ribosomal protein S6 kinase, polypeptide 5* | NM_001108048 | 0.0385 | 50.52 | -1.05 |
| Plekhm1 | *Pleckstrin Homology Domain Containing, Family M (With RUN Domain) Member 1* | NM_001009677 | 0.0295 | 47.72 | -1.05 |
| Prkaa1 | *Protein Kinase, AMP-Activated, Alpha 1 Catalytic Subunit* | NM_019142 | 0.0111 | 35.28 | -1.05 |
| Sos2 | *son of sevenless homolog 2 (Drosophila)* | NM_001135561 | 0.0199 | 43.10 | -1.05 |
| Folh1 | *folate hydrolase 1* | NM_057185 | 0.0265 | 47.72 | -1.05 |
| Ankrd46 | *ankyrin repeat domain 46* | NM_001013948 | 0.0474 | 54.56 | -1.05 |
| Mier1 | *Mesoderm Induction Early Response 1, Transcriptional Regulator* | NM_001131012 | 0.0471 | 54.56 | -1.05 |
| P4htm | *prolyl 4-hydroxylase, transmembrane* | ENSRNOT00000027466 | 0.0424 | 50.52 | -1.05 |
| Elovl5 | *ELOVL family member 5, elongation of long chain fatty acid* | NM_134382 | 0.0447 | 50.52 | -1.05 |
| Syncrip | *Synaptotagmin Binding, Cytoplasmic RNA Interacting Protein* | NM_001047916 | 0.0377 | 50.52 | -1.05 |
| Osbpl2 | *oxysterol binding protein-like 2* | NM_001013079 | 0.0264 | 47.72 | -1.05 |
| Rpl3 | *ribosomal protein L3* | NM_198753 | 0.0200 | 43.10 | -1.05 |
| Zdhhc17 | *zinc finger, DHHC-type containing 17* | NM_001039340 | 0.0299 | 47.72 | -1.05 |
| Slc7a5 | *solute carrier family 7 (cationic amino acid transporter, Large neutral amino acids transporter)* | NM_017353 | 0.0427 | 50.52 | -1.05 |
| Chst2 | *carbohydrate sulfotransferase 2* | ENSRNOT00000056689 | 0.0313 | 47.72 | -1.05 |
| Wdtc1 | *WD and tetratricopeptide repeats 1* | NM_001107908 | 0.0257 | 43.10 | -1.05 |
| Mcart1 | *mitochondrial carrier triple repeat 1* | NM_001024785 | 0.0334 | 47.72 | -1.05 |
| Nrsn1 | *neurensin 1* | NM_001106109 | 0.0245 | 43.10 | -1.05 |
| LOC685152 | *similar to Probable phospholipid-transporting* | ENSRNOT00000056606 | 0.0095 | 32.49 | -1.05 |
| Zfp148 | *zinc finger protein 148* | NM_031615 | 0.0021 | 23.21 | -1.05 |
| Tmem178 | *transmembrane protein 178* | NM_001004282 | 0.0486 | 54.56 | -1.05 |
| H2rgscomt  afy | *H2A histone family, member Y* | NM_017182 | 0.0382 | 50.52 | -1.05 |
| Hif1a | *Hypoxia Inducible Factor 1, Alpha Subunit (Basic Helix-Loop-Helix Transcription Factor)* | NM_024359 | 0.0256 | 43.10 | -1.05 |
| Glud1 | *glutamate dehydrogenase 1* | NM_012570 | 0.0143 | 37.73 | -1.05 |
| Mt3 | *metallothionein 3* | NM_053968 | 0.0470 | 54.56 | -1.05 |
| Mtch1 | *mitochondrial carrier homolog 1 (C. elegans)* | NM_001100833 | 0.0453 | 54.56 | -1.05 |
| Rpl31 | *ribosomal protein L31* | NM_022506 | 0.0278 | 47.72 | -1.05 |
| Mtmr12 | *myotubularin related protein 12* | NM_001012077 | 0.0289 | 47.72 | -1.05 |
| Ttll7 | *tubulin tyrosine ligase-like family, member 7* | ENSRNOT00000055318 | 0.0382 | 50.52 | -1.05 |
| Rpl31 | *ribosomal protein L31* | NM_022506 | 0.0368 | 50.52 | -1.04 |
| Ctcf | *CCCTC-binding factor (zinc finger protein)* | NM_031824 | 0.0476 | 54.56 | -1.04 |
| Arl1 | *ADP-ribosylation factor-like 1* | NM_022385 | 0.0381 | 50.52 | -1.04 |
| Kctd3 | *potassium channel tetramerisation domain containing 3* | NM_001107199 | 0.0287 | 47.72 | -1.04 |
| Tcf25 | *transcription factor 25 (basic helix-loop-helix)* | ENSRNOT00000046155 | 0.0025 | 23.21 | -1.04 |
| Zc3h15 | *zinc finger CCCH-type containing 15* | NM_001010963 | 0.0342 | 47.72 | -1.04 |
| Fth1 | *ferritin, heavy polypeptide 1* | NM_012848 | 0.0040 | 25.15 | -1.04 |
| Rpl31 | *ribosomal protein L31* | NM_022506 | 0.0421 | 50.52 | -1.04 |
| Zfp280d | *zinc finger protein 280D* | NM_001108165 | 0.0385 | 50.52 | -1.04 |
| Map4 | *microtubule-associated protein 4* | NM_001024278 | 0.0401 | 50.52 | -1.04 |
| Fth1 | *ferritin, heavy polypeptide 1* | NM_012848 | 0.0062 | 27.96 | -1.04 |
| Dcun1d4 | *DCN1, Defective In Cullin Neddylation 1, Domain Containing 4* | NM_001108359 | 0.0474 | 54.56 | -1.04 |
| Otud4 | *OTU domain containing 4* | NM_001191700 | 0.0112 | 35.28 | -1.04 |
| Ddhd1 | *DDHD domain containing 1* | NM_001033066 | 0.0124 | 35.28 | -1.04 |
| Ivns1abp | *influenza virus NS1A binding protein* | NM_001047085 | 0.0370 | 50.52 | -1.04 |
| Ywhab | *tyrosine 3-monooxygenase* | NM_019377 | 0.0447 | 50.52 | -1.04 |
| Stxbp1 | *syntaxin binding protein 1* | NM_013038 | 0.0037 | 25.15 | -1.04 |
| Camk2b | *calcium* | NM_001042354 | 0.0361 | 50.52 | -1.04 |
| Ostm1 | *osteopetrosis associated transmembrane protein 1* | NM_001029925 | 0.0420 | 50.52 | -1.04 |
| Rps26 | *ribosomal protein S26* | NM_013224 | 0.0149 | 37.73 | -1.04 |
| Znf644 | *zinc finger protein 644* | NM_001139484 | 0.0067 | 29.67 | -1.04 |
| Phf19 | *PHD finger protein 19* | NM_001106570 | 0.0450 | 54.56 | -1.04 |
| Add2 | *adducin 2 (beta)* | NM_001109880 | 0.0439 | 50.52 | -1.04 |
| Pdpk1 | *3-phosphoinositide dependent protein kinase-1* | NM_031081 | 0.0114 | 35.28 | -1.04 |
| Asrgl1 | *asparaginase like 1* | NM_145089 | 0.0284 | 47.72 | -1.04 |
| Atp2c1 | *ATPase, Ca++ transporting, type 2C, member 1* | NM_131907 | 0.0233 | 43.10 | -1.04 |
| RGD1564200 | *similar to hypothetical protein MGC34646* | NM_001108969 | 0.0261 | 47.72 | -1.04 |
| Cacnb1 | *calcium channel, voltage-dependent, beta 1 subunit* | NM_017346 | 0.0298 | 47.72 | -1.04 |
| Sfrs2 | *splicing factor, arginine* | NM_001009720 | 0.0280 | 47.72 | -1.04 |
| Scnm1 | *sodium channel modifier 1* | NM_001107696 | 0.0332 | 47.72 | -1.04 |
| Slc6a1 | *solute carrier family 6 (neurotransmitter transporter, GABA, GAT-1)* | NM_024371 | 0.0291 | 47.72 | -1.04 |
| Mkln1 | *muskelin 1, intracellular mediator containing kelch motifs* | NM_031359 | 0.0150 | 37.73 | -1.04 |
| Sept3 | *septin 3* | NM_019375 | 0.0449 | 54.56 | -1.04 |
| Hspa8 | *heat shock protein A8* | NM_024351 | 0.0121 | 35.28 | -1.04 |
| Eno2 | *enolase 2, gamma, neuronal* | NM_139325 | 0.0177 | 40.47 | -1.04 |
| Prkar1b | *Protein Kinase, CAMP-Dependent, Regulatory, Type I, Beta* | NM_001033679 | 0.0433 | 50.52 | -1.04 |
| Rpl29 | *ribosomal protein L29* | NM_017150 | 0.0375 | 50.52 | -1.04 |
| Rufy3 | *RUN and FYVE domain containing 3* | NM_001025127 | 0.0060 | 27.96 | -1.04 |
| RGD1304878 | *similar to 2410024A21Rik protein* | ENSRNOT00000006131 | 0.0246 | 43.10 | -1.04 |
| Mars | *methionine-tRNA synthetase* | NM_001127659 | 0.0371 | 50.52 | -1.04 |
| Clip3 | *CAP-GLY domain containing linker protein 3* | NM_001107501 | 0.0455 | 54.56 | -1.04 |
| Dnajc7 | *DnaJ (Hsp40) homolog, subfamily C, member 7* | NM_213625 | 0.0307 | 47.72 | -1.04 |
| Mfn2 | *mitofusin 2* | NM_130894 | 0.0411 | 50.52 | -1.04 |
| Ppp1r13b | *protein phosphatase 1, regulatory (inhibitor) subunit 13 beta* | NM_001108062 | 0.0359 | 50.52 | -1.04 |
| Mapk8ip2 | *mitogen-activated protein kinase 8 interacting protein 2* | ENSRNOT00000055792 | 0.0482 | 54.56 | -1.04 |
| Chn1 | *chimerin (chimaerin) 1* | NM_032083 | 0.0465 | 54.56 | -1.04 |
| Epb4.1l1 | *erythrocyte protein band 4.1-like 1* | NM_021681 | 0.0436 | 50.52 | -1.03 |
| Txnl1 | *thioredoxin-like 1* | NM_080887 | 0.0421 | 50.52 | -1.03 |
| Actg1 | *actin, gamma 1* | NM_001127449 | 0.0432 | 50.52 | -1.03 |
| Atp1a2 | *ATPase, Na+* | NM_012505 | 0.0071 | 29.67 | -1.03 |
| Rpl19 | *ribosomal protein L19* | NM_031103 | 0.0380 | 50.52 | -1.03 |
| Actg1 | *actin, gamma 1* | NM_001127449 | 0.0361 | 50.52 | -1.03 |
| Gabbr1 | *gamma-aminobutyric acid (GABA) B receptor 1* | NM_031028 | 0.0265 | 47.72 | -1.03 |
| Actg1 | *actin, gamma 1* | NM_001127449 | 0.0462 | 54.56 | -1.03 |
| Gorasp2 | *golgi reassembly stacking protein 2* | NM_001007720 | 0.0484 | 54.56 | -1.03 |
| Atp1b2 | *ATPase, Na+/K+ Transporting, Beta 2 Polypeptide* | NM_012507 | 0.0354 | 50.52 | -1.03 |
| Mapk8ip3 | *mitogen-activated protein kinase 8 interacting protein 3* | NM_001100673 | 0.0455 | 54.56 | -1.03 |
| Lrrc4b | *leucine rich repeat containing 4B* | ENSRNOT00000026259 | 0.0271 | 47.72 | -1.03 |
| Actg1 | *actin, gamma 1* | NM_001127449 | 0.0492 | 54.56 | -1.03 |
| Smap2 | *small ArfGAP2* | NM_001100669 | 0.0380 | 50.52 | -1.03 |
| Dpp8 | *dipeptidylpeptidase 8* | NM_001108159 | 0.0304 | 47.72 | -1.02 |
| Clptm1 | *cleft lip and palate associated transmembrane protein 1* | NM_001106232 | 0.0293 | 47.72 | -1.02 |
| Olr1459 | *olfactory receptor 1459* | NM_001000770 | 0.0001 | 9.75 | 1.11 |
| Csrp2 | *cysteine and glycine-rich protein 2* | NM_177425 | 0.0001 | 9.75 | 1.15 |
